# Targeting the Tumor Extracellular Matrix by the Natural Molecule 4-Methylumbelliferone: A Complementary and Alternative Cancer Therapeutic Strategy

**DOI:** 10.3389/fonc.2021.710061

**Published:** 2021-10-04

**Authors:** Daiana L. Vitale, Antonella Icardi, Paolo Rosales, Fiorella M. Spinelli, Ina Sevic, Laura D. Alaniz

**Affiliations:** ^1^ Laboratorio de Microambiente Tumoral, Centro de Investigaciones Básicas y Aplicadas (CIBA), Universidad Nacional del Noroeste de la Provincia de Buenos Aires, Junin, Argentina; ^2^ Centro de Investigaciones y Transferencia del Noroeste de la Provincia de Buenos Aires (CITNOBA), Universidad Nacional del Noroeste de la Provincia de Buenos Aires (UNNOBA), Universidad Nacional de San Antonio de Areco (UNSAdA), Consejo Nacional de Investigaciones Científicas y Técnicas (CONICET), Pergamino, Argentina; ^3^ Centre de Recherche en Cancérologie et Immunologie Nantes Angers (CRCINA), Inserm, Centre National de la Recherche Scientifique (CNRS), Université de Nantes, Nantes, France

**Keywords:** 4-methylumbelliferone, hyaluronan, extracellular matrix, cancer, antitumoral action

## Abstract

In antineoplastic therapy, one of the challenges is to adjust the treatment to the needs of each patient and reduce the toxicity caused by conventional antitumor strategies. It has been demonstrated that natural products with antitumoral properties are less toxic than chemotherapy and radiotherapy. Also, using already developed drugs allows developing substantially less costly methods for the discovery of new treatments than traditional drug development. Candidate molecules proposed for drug repositioning include 4-methylumbelliferone (4-MU), an orally available dietetic product, derivative of coumarin and mainly found in the plant family Umbelliferae or Apiaceae. 4-MU specifically inhibits the synthesis of glycosaminoglycan hyaluronan (HA), which is its main mechanism of action. This agent reduces the availability of HA substrates and inhibits the activity of different HA synthases. However, an effect independent of HA synthesis has also been observed. 4-MU acts as an inhibitor of tumor growth in different types of cancer. Particularly, 4-MU acts on the proliferation, migration and invasion abilities of tumor cells and inhibits the progression of cancer stem cells and the development of drug resistance. In addition, the effect of 4-MU impacts not only on tumor cells, but also on other components of the tumor microenvironment. Specifically, 4-MU can potentially act on immune, fibroblast and endothelial cells, and pro-tumor processes such as angiogenesis. Most of these effects are consistent with the altered functions of HA during tumor progression and can be interrupted by the action of 4-MU. While the potential advantage of 4-MU as an adjunct in cancer therapy could improve therapeutic efficacy and reduce toxicities of other antitumoral agents, the greatest challenge is the lack of scientific evidence to support its approval. Therefore, crucial human clinical studies have yet to be done to respond to this need. Here, we discuss and review the possible applications of 4-MU as an adjunct in conventional antineoplastic therapies, to achieve greater therapeutic success. We also describe the main proposed mechanisms of action that promote an increase in the efficacy of conventional antineoplastic strategies in different types of cancer and prospects that promote 4-MU repositioning and application in cancer therapy.

## 1 Introduction

Natural products derived from plants have been extensively used for thousands of years. However, to guarantee their correct application and safety, their benefits should be thoroughly investigated through both basic and clinical studies. Although the World Health Organization (WHO) has established the operational guide to use and conduct clinical studies of these products, rules and regulations depend on the region or country. Several products that contain active principles from plant extracts are already included in the health system, but their percentages in the prescription depend on the authorization by entities such as the European Medicines Agency (EMA) or the Food and Drug Administration (FDA). For example, in an analysis made of prescriptions dispensed from community pharmacies in the USA between 1959 and 1980, 25% were products derived from plants (1). Among these herbal-derived products are coumarins, whose name originated from the fact that they were first found in the seed of the tree *Dipteryx odorata* of the family *Fabaceae*, commonly known as “cumaru” or “kumaru” in Central and South America (2). Coumarin derivatives are currently extracted from many plants across continents and are found in high levels in fruits, roots, stems and leaves (3). It has been described that coumarin and its derivatives have diverse biological effects, acting as anti-inflammatory (4), anticoagulant (5), antiviral (6), fungicidal (7) and antitumor agents (8). Chemically, they are benzo-α-pyrones (IUPAC nomenclature: 2H-chromen-2-one), which consist of a benzene ring joined to a pyrone ring. Among coumarin derivatives is 4-methylumbelliferone (4-MU), considered to belong to the group of simple coumarins (9). 4-MU is hydroxylated in position seven, known as umbelliferone, and methylated in position four (IUPAC nomenclature: 7-hydroxy-4-methylcoumarin), and also known by the international nonproprietary or generic name: hymecromone. The information provided in the National Center for Advancing Translational Sciences (NCATS) Inxight portal Drugs indicates that this substance is approved in Europe and Asia to treat biliary spasm and is used orally as a choleretic and antispasmodic drug and as a standard for the fluorometric determination of enzyme activity (https://drugs.ncats.io/).

Umbelliferones are widely distributed among the plant families *Rutaceae*, *Umbelliferae* (celery, anise) and *Asteraceae* (chamomile) (1). However, since these compounds are not easily extracted from plants, they are synthesized using the “Pechmann” condensation reaction of resorcinol and formyl acetic acid (10). Our interest in these molecules lies on their mechanism of action. In particular, 4-MU is able to inhibit hyaluronan (HA) synthesis since the active glucuronidation of 4-MU depletes the cellular UDP-glucuronic acid (UDP-GlcUA) pool necessary for HA synthesis. It has also been determined that 4-MU downregulates the mRNA levels of HA synthases (HAS) (11). Since HA is an important extracellular glycosaminoglycan, able to modulate tumor behavior (12), 4-MU can be considered as a drug with antitumor action. In addition, some reports have demonstrated that its therapeutic action in pathological conditions relies on more than just its effects on HA synthesis (13, 14). However, it is still necessary to deepen the knowledge on this mechanism of action. As mentioned, 4-MU depletes the UDP-GlcUA pool, whose synthesis is dependent on glucose metabolism, thus affecting the cellular energetic state (15). Besides, several metabolic routes that use UDP-GlcUA, such as conjugation reaction, which allows inactivation of other metabolites, could be affected.

Thus, in this review, we discuss the tumor process that might be modulated by 4-MU, focusing on the type of tumor as well as on its action on different tumor-associated cells besides the tumor cell itself.

## 2 Pharmacological Aspects

### 2.1 4-MU Metabolism

4-MU metabolism gives rise to a limited number of metabolites however the metabolites that are produced depend on the species (3). Specifically, 4-MU is metabolized mainly by glucuronyltransferases to a glucuronide conjugate in phase II reactions, transforming it into 4-methylumbelliferone-beta-D-glucuronide (4-MUG) (16). 4-MU, like other coumarins, is insoluble in water, and since it is not a polar molecule, it can cross the lipidic intestinal barrier easily, allowing its complete absorption when orally administered, finally binding to plasma protein, which allows it to adequately reach the tissues (3). It has a short half-life and low bioavailability and is excreted primarily in urine (17). Besides, the methyl group in position four offers 4-MU several advantages over the other derived molecules, such as lower toxicity, since it prevents its metabolism to the mutagenic 3,4-coumarin epoxide by the action of liver cytochrome P450 enzymes (18), and lower anticoagulant effect compared to dicoumarol or warfarin. Thus, products containing 4-MU are available in the USA and Europe as dietary supplements (Heparvit^®^, Heparmed^®^, DetoxPro^®^). Besides, a clinical trial in the USA in patients with chronic hepatitis B and C (ClinicalTrials.gov identifier NCT00225537) has also demonstrated that 4-MU is safe, reaching phase II of the study in 2007, although complete results are not published yet. The dose ranges used in humans are between 8 and 7000 mg/day (19), being several times higher than the acceptable daily intake in food and cosmetic products, which is 0.06 mg/kg/day (20). However, no mutagenic or genotoxic effects have been observed (21). This makes it an interesting compound to consider for use in several diseases and propose its repositioning in cancer, since positive responses have been observed even in advanced stages of this disease (22).

Based on studies in rats, which are poor models to compare with humans for this particular type of metabolism, the FDA classified coumarins as toxic compounds (9). However, as compared with their hepatotoxicity in rats and mice (23), studies carried out in humans have shown little evidence of liver dysfunction (3). Moreover, as compared with other coumarin derivatives, 4-MU has been safely used in liver therapy as a choleretic and spasmolytic, improving liver detoxification systems through increased bile production (24). In humans, 4-MU is consumed at a dose of 1500 to 2200 mg/day as a choleretic, and, in several cancer models in mice, it has shown antitumor activity in doses of 1000 to 3000 mg/kg, being the maximal tolerated dose 2300 to 7200 mg/kg (3). Thus, taking into account this pharmacological aspect of 4-MU, it is possible to suggest that, in combination with other cancer chemotherapeutic drugs, these doses could be lower without loss of their effectiveness, providing additive or synergistic effects, as will be discussed below.

### 2.2 Differential Pharmacological Effects of 4-MU

Regarding the undesirable pharmacological effects of 4-MU, García-Vilas et al. observed that it could show a potent antiangiogenic effect by inducing the inhibition of HA synthesis and that since HA is a normal constituent of the extracellular matrix (ECM) in several tissues in humans, its longtime use might cause systemic damage (25). In the context of cancer, it would be considered that 4-MU should be used at similar time and in combination with a chemotherapy protocol. Therefore, tissues that have active HA synthesis could be affected during chemotherapy treatment. Besides, due to the current difficulty of deliver the drug in a tumor-specific manner, the time schedule during cancer treatment must be carefully studied in human patients. In an atherosclerosis mouse model, Nagy et al. found that 4-MU alters the normal vascular endothelial glycocalyx, favoring its progression (26). This also suggests that this compound could induce side-effects like cardiovascular alterations, and therefore the correct dose and treatment time should be analyzed in different contexts to reduce potential adverse effects. However, experiments at our lab support the hypothesis that, in a context where HA is overproduced, 4-MU could have therapeutic effect. In hepatocellular cell lines with different levels of HA production, we observed that significant antiproliferative or apoptotic effects were detected only in cells with high HA levels (27). In fact, 4-MU treatment has been found to be beneficial for pathologies with high level or dysregulated synthesis of HA like endometriosis (28), where the adherence of menstrual CD44-expressing endometrial cells to mesothelial cells *via* binding to HA is involved in endometriotic lesions, or autoimmune diseases, such as rheumatoid arthritis, type 1 Diabetes or multiple sclerosis, where the chronic inflammation state is associated with abnormal deposition of HA in the synovial compartment, pancreatic islets and spaces between myelinated axons, respectively (29).

In the next section, we will discuss the antitumor effects of 4-MU in different types of cancers, which is the focus of this review.

## 3 Antitumoral Effects of 4-MU Treatment in Different Types of Cancer

In several human cancers, HA concentration is increased (30, 31) and it is well known that a HA-rich stroma has an active role in the tumor microenvironment, promoting tumor development, angiogenesis, metastasis (32, 33), and drug resistance (34), and even acting as an immune-regulatory factor (35). Therefore, targeting HA synthesis by 4-MU represents a specific therapeutic approach to control HA levels in the cancer cell stroma. Several reports have shown that 4-MU inhibits the proliferation, migration, and invasion of multiple cancer types, both *in vitro* and *in vivo*, by a mechanism dependent on the inhibition of HA synthesis (**Table 1**), which will be the mechanism mainly discussed, although independent mechanisms will also be reviewed.

**Table 1:** Effect of 4-MU treatment in different types of tumors.

Tumor	Effect of 4-MU treatment (*in vitro* and/or *in vivo*)	References
Colon carcinoma	*Higher expression of antiangiogenic factors*	(36)
	*Higher migration rates of cytotoxic T lymphocytes*	
	*Reduction of tumor interstitial pressure*	
Pancreatic cancer	*Suppressed cell proliferation, migration and invasion*	(37–42)
	*Increased apoptosis*	
	*Alterations in intercellular spaces*	
	*Decreased liver metastasis*	
	*Potentiated effect of gemcitabine and 5-fluoruracil*	
	*Enhanced cytotoxic effect of T lymphocytes*	
Prostate cancer	*Inhibited proliferation, motility and invasion*	(43, 44)
	*Higher apoptosis*	
	*Decreased tumor growth and microvessel formation*	
Ovarian cancer	*Inhibition of cell migration, proliferation and invasion*	(45–47)
	*Decreased tumor growth*	
Breast cancer	*Inhibition of the proliferation of human breast carcinoma cells*	(48–51)
	*Decreased cell motility, invasion and proliferation*	
	*Decreased incidence of metastasis and growth of CSC in the bone*	
Hepatocellular carcinoma	*Inhibition of cancer stem cell properties*	(27, 52, 53)
	*Reduction of liver fibrosis and impairment of tumor growth by reduction of proangiogenic factors*	
Bone-derived cancer	*Osteosarcoma:*	(54–57)
	*Inhibition of cell proliferation, migration and invasion.*	
	*Reduced lung metastasis*	
	*Chondrosarcoma:*	
	*Suppression of cell proliferation, migration and invasion*	
	*Inhibition of local tumor growth*	
	*Fibrosarcoma:*	
	*Positive effect on the sensitivity of cells to radiotherapy*	
Melanoma	*Inhibition of cell adhesion and locomotion*	(58–60)
	*Suppression of liver metastasis*	
	*Positive effect on the sensitivity of cells to vemurafenib*	
Chronic myeloid leukemia	*Induction of apoptosis in vitro and in vivo*	(61–64)
	*Reduced tumor growth*	
	*Sensitization of CML cells to doxorubicin and vincristine*	
Glioblastoma	*Decreased cell migration and proliferation*	(65–67)
	*Induction of apoptosis*	
	*Sensitization of glioblastoma cells to temozolomide*	

### 3.1 Colorectal Carcinoma

Colorectal cancer (CRC), one of the most observed types of tumor worldwide, presents treatment limitations due to the necessity of surgical treatment and the high rates of metastasis and mortality (68). For this reason, it is one of the main targets of the investigation about alternative therapies that seek to control tumor spread and reduce mortality. In this sense, several scientific reports have demonstrated the specific role of 4-MU in CRC. In colon cancer cells, Heffler et al. *showed that the inhibition of* the inhibition of HAS and HA decreases tumor growth and increase apoptosis in a dose-dependent manner (69). Similarly, in the HCT-8 cell line, Wang et al. showed that 4-MU can effectively reduce 2D and 3D proliferation as well as cell motility and that this effect could be reversed by addition of exogenous HA, indicating that the reduction of HA production in cancer cells could inhibit tumor growth and metastasis (70). In another metastatic CRC cell line, SW620, Heffler et al. also found that *in vitro* treatment with 4-MU significantly reduced cell viability (69). Besides, based on the fact that HA and focal adhesion kinase (FAK) signaling are associated with the promotion of tumorigenesis, these authors observed that 4-MU could act synergistically during FAK inhibition (69). Also, in CT26 CRC cells, Malvicini et al. observed that 4-MU significantly reduced HA synthesis without affecting their viability and that, in an *in vivo* mouse model, the reduction of HA by 4-MU treatment reduced tumor interstitial pressure without affecting tumor growth (36). However, in this model, the authors also found that 75% of mice treated with 4-MU in combination with cyclophosphamide and IL-12 showed tumor regression (36). This triple combination induced the production of antiangiogenic factors and increased the migration of cytotoxic T lymphocytes in tumors, showing that tumor microenvironment remodeling and reduction of HA synthesis increase the efficacy of anticancer immunotherapies combined with chemotherapy agents (36).

These reports indicate that, in CRC models, 4-MU exerts its action by inhibiting HA synthesis, but the impact of this inhibition could be associated or not with the modulation of tumor cell survival, suggesting that it affects both tumor cells and the tumor microenvironment.

### 3.2 Pancreatic Cancer

Pancreatic ductal adenocarcinoma (PDAC) is the most malignant of all solid cancers because of the difficulties in early diagnosis and the poor response to chemotherapy (37). PDAC has an abundant volume of stroma composed of large amounts of HA (30, 71). It has been demonstrated that, in this type of cancer, 4-MU inhibits HA synthesis, thus affecting tumor cell behavior (38). In pancreatic cancer cells, Nagase et al. first determined that 4-MU suppressed cell proliferation and invasion and increased apoptosis by inhibiting HA production (37). Then, in an *in vivo* mouse model of PDAC, these authors found that 4-MU treatment suppressed HA accumulation in pancreatic tumor tissue and improved survival rate (37). To better understand tumor microenvironment interactions, Cheng et al. studied this inhibition in PDAC Panc-1 cells co-cultured with stromal fibroblasts (39). Specifically, they. found that 4-MU inhibited the enhanced migration of PDAC cells in response to tumor-stromal interactions with fibroblasts (39). In addition, Nakazawa et al. showed that 4-MU inhibited HA synthesis and the formation of the pericellular HA coat in KP1-NL pancreatic cells and decreased liver metastases *in vivo* (40). In another human pancreatic cancer cell-bearing mouse model, Yoshida et al. observed a decrease in tumor volume and a significant reduction of the intratumoral HA amount (41). Besides, histological analysis by electron microscopy revealed that 4-MU altered the intercellular space, causing it to become less cohesive and more permissive to drug delivery, indicating that this could be a promising combination with chemotherapy agents, improving their effects (41). In fact, several reports have indicated the potential role of 4-MU as a co-adjuvant during the chemotherapeutic treatment of this cancer. In this regards, Nagase et al. found that *in vivo* co-administration of 4-MU and the chemotherapeutic drug gemcitabine to tumor-inoculated mice decreased the size of primary and metastatic tumors more than gemcitabine alone (37). By combining 5-fluorouracil with 4-MU treatment in an *in vivo* pancreatic cancer model, Yoshida et al. found similar results, where 4-MU potentiated the effects of 5-fluorouracil by sensitizing tumor cells to its cytotoxic action (42). Also, the role of 4-MU as a modulator of immunotherapy strategy during PDAC has been recently determined. Suto et al., for example, have recently shown that 4-MU inhibited PDAC cell proliferation and HA synthesis in four different PDAC cell lines, and enhanced γδ T-cell-rich peripheral blood mononuclear cell-mediated cytotoxicity against pancreatic cells (72). These authors found the same results *in vivo*, where 4-MU reduced intratumor HA deposition and promoted infiltration of transferred γδ T-cells into tumor tissue, and consequently suppressed tumor growth (72). These data indicate that 4-MU inhibits HA synthesis and reduces the amount of HA in the ECM of prostate cancer, thus affecting tumor cell behavior and its response to chemo- or immunotherapy.

### 3.3 Prostate Cancer

Some researchers have proposed that, in prostate cancer, 4-MU acts as a regulator of HA synthesis and angiogenesis. Lokeshwar et al., for example, studied the effects of 4-MU on different prostate cancer cell lines and demonstrated that 4-MU inhibited proliferation, motility, and invasion and increased apoptosis (43, 44). Besides, in a mouse model of prostate cancer, these authors observed that oral administration of 4-MU significantly decreased transgenic adenocarcinoma and PC3-ML tumor growth without organ toxicity or changes in serum chemistry or body weight. They also found that tumors from 4-MU–treated animals showed reduced microvessel density and downregulated HA receptors, Akt signaling and β-catenin activation (43, 44). Although not many reports have evaluated 4-MU as a modulator of prostate cancer behavior, these studies, together with other studies analyzing the effect of 4-MU in other types of tumors, reinforce the inhibitory role of 4-MU in prostate cancer growth with an anti-angiogenic potential. Therefore, these data open up new avenues of investigation of the effect of this natural molecule on pancreatic cancer and its possible therapeutic applications.

### 3.4 Ovarian Cancer

Ovarian cancer is one of the most frequent gynecological pathologies in adult women. It has a high mortality rate since it metastasizes early and quickly, presenting high resistance to chemotherapy (45, 73). Importantly, high levels of HA have been detected in histological samples from tumor and metastatic lesions derived from patients with epithelial ovarian cancers with worse prognostics, suggesting that this molecule could be considered a therapeutic target (46). Thus, many studies are currently assessing the ability of natural products as 4-MU to induce ovarian cancer cell death and complement the antitumor treatment. One of the first studies performed for Kultti et al. showed in SKOV-3 ovarian cancer cells determined that 4-MU inhibits HA synthesis and produces large quantities of 4-MU-glucuronide *in vitro*, depleting the cellular UDP-GlcUA source (11). The inhibitory effect of 4-MU has also been observed in the down-regulation of HAS3 expression (11). In addition, Anttila et al. found that the reduction of the HA-pericellular coat was related to the inhibition of cell migration, proliferation and invasion (46). Extending the studies on ovarian cancer, Tamura et al. demonstrated the effect of 4-MU on HRA human ovarian serous adenocarcinoma cells, using *in vitro* assays and an *in vivo* rat peritoneal carcinoma model (47). These authors found that 4-MU inhibited ovarian cancer cell proliferation in a dose-dependent manner *in vitro*, but also found non-inhibitory effects of 4-MU on cell invasion and migration (47). In their *in vivo* experiments they found that 4-MU administration inhibited the growth of peritoneal tumors and significantly prolonged rat survival (47). Recently, An et al. have determined the molecular mechanisms associated with the inhibitory effect of 4-MU on ES2 and OV90 epithelial ovarian cancer cells (45). Specifically, they observed a decrease in cell proliferation and cell arrest in the G2/M phase of the cell cycle, which defines lower cell division rates. They also found that 4-MU interfered with calcium homeostasis, induced endoplasmic reticulum stress, inhibited AKT and S6 phosphorylation, and increased MAPK phosphorylation (45).

Certain ovarian cell carcinomas show a spherule-like mucoid stroma with a hollow acellular space. Despite the absence of stromal cells, both the mucoid stroma and hollow spheroids contain abundant ECM, mainly composed of HA, which plays a crucial role in the formation of those structures and in tumor progression. In this sense, Kato et al. determined that after 4-MU treatment of HAC-2 ovarian cancer cells, HA synthesis was inhibited and consequently, the spherule-like accumulation of HA and hollow spheroids were not observed (74). These authors determined that the inhibition of HA synthesis was associated with the reduction of cell growth (74).

All these reports indicate that tumor-derived HA is essential for the regulation of cell growth, migration and invasion ability of ovarian clear cell carcinoma. Thus, the inhibition of HA synthesis could be a potential adjunctive therapy, avoiding the interaction of this molecule with its receptors, like CD44, and in turn blocking the signaling that allows tumor dissemination in this type of cancer. However, it has been observed that 4-MU effect could also be independent of the modulation of HA expression, affecting other tumor signals besides HA-CD44, a fact that also supports its therapeutic use.

### 3.5 Breast Cancer

Breast cancer is one of the most frequently diagnosed cancers in women and is considered to have a high phenotypic diversity, which heavily influences the progression and outcome of the treatment. In this sense, three receptors are frequently analyzed for the correct treatment decision: the estrogen receptor (ER), the progesterone receptor (PR) and the human epidermal growth factor receptor 2 (HER2) (75). Some reports have shown that 4-MU treatment leads to changes in the proliferative phenotype of ER^-^ and ER^+^ cells (48, 75). Karalis et al., for example, found that 4-MU treatment led to a reduction in cell proliferation in both cell types, which, in ER+ cells, was more pronounced after 2 days, and in ER- cells much faster on the first day of treatment (48). This quicker reaction of ER- cells to lower concentrations of 4-MU than ER+ cells could indicate a stronger susceptibility of these cells to low 4-MU concentrations showed that 4-MU inhibits proliferation of human breast carcinoma cells in different cell lines, like T-47D (ER^+^PR^+^HER2^-^) and MDA-MB-231 (ER^-^PR^-^HER2^-^) cells (48). Additionally, these authors showed that low levels of HA and glucose in the tumor microenvironment could increase the sensitivity of breast cancer cells to 4-MU treatment and thus inhibit cell proliferation more strongly (48).

In breast cancer cell lines with highly invasive character, such as MDA-MB-231 cells. Urakawa et al. demonstrated that 4-MU suppresses HA synthesis and accumulation probably due to the suppression of HAS2 expression, which could in turn lead to lower cell motility, invasion and proliferation (49). By using 4-MU to inhibit HA synthesis in breast cancer cells, Brett et al. suggested that a decrease in pericellular matrix formation is correlated with decreased invasiveness, and proposed that a reduction in HA synthesis could inhibit the formation of the pericellular matrix and provide a good strategy for inhibition of metastatic progression (50). Also, Kultti et al. showed that 4-MU inhibits migration of the non-invasive MCF-7 (ER^+^PR^+^HER2^-^) breast cancer cells and that the growth of these cells is sensitive to 4-MU, being almost completely blocked by high concentrations of the drug (11). These authors also showed that 4-MU inhibits HA by reduction of the cellular HAS substrate UDP-GlcUA and that in MCF-7 cells this reduction was dose-sensitive, with less pronounced response at higher doses, while MDA-MB-361 (ER^+^PR^-^HER2^+^) cells lost most of their UDP-GlcUA at higher doses of 4-MU (11).

To form metastasis, metastatic tumor cells usually move into a specific organ. In particular, breast cancer preferentially metastasizes to the bone and lungs. Okuda et al. showed that cancer stem cells (CSCs) from a metastatic breast tumor show considerably higher tumorigenic and metastatic capability than CSCs from a low-metastatic tumor and indicate that HAS2 is essential to provide CSCs with a metastatic phenotype (51). These authors proposed that 4-MU, due to the specific inhibition of HA by affecting HAS2 activity, can considerably suppress the incidence of metastasis and growth of CSCs in the bone (51).

Thus, the above-mentioned reports indicate that 4-MU could be beneficial to treat breast cancer, although the sensitivity of tumor cells and the response to this drug will depend on the hormonal receptor status. Interestingly, this opens a line of investigation that could associate ECM remodeling by 4-MU with the signal mediated by progestogens in breast cancer. On the other hand, it is important to highlight that, due to its ability to modulate the phenotype of CSCs, 4-MU has a great therapeutic potential and could help to control tumor resistance.

### 3.6 Hepatocellular Carcinoma

Hepatocellular carcinoma (HCC) is a tumor that frequently occurs in the inflammatory microenvironment, usually as a reaction process that arises in response to chronic injuries, like chronic hepatitis C and B virus infection or alcohol abuse (76). Regardless of the etiology, in chronic liver disease, the ECM components, like HA and collagen, deposit in the liver, depending on the level of fibrosis progression. For this reason, the level of HA could be used as a biomarker to assess the stage of liver fibrosis (77). In high-HA-producing murine Hepa129 cells and in medium-HA-producing human Hep3B cells, Piccioni et al. showed that 4-MU inhibited proliferation and induced apoptosis (78). Contrarily, in human low-HA-producing Huh7 cells, these authors observed partial resistance to 4-MU treatment (78). These results show that the mechanism of 4-MU action in HCC is highly dependent on HA levels (78). It has also been demonstrated that 4-MU, by inhibiting HA, could reduce liver fibrosis and diminish tumor growth by reduction of proangiogenic factors, like VEGF and CXCL12, and also by reduction of IL-6 production in the liver tumor microenvironment (27). Some reports have shown that 4-MU inhibits the properties of CSCs by the inhibition of HA, accompanied by a reduction of CSC markers, like transmembrane glycoproteins CD44 and CD133, as well as CD90 and EpCAM cells, indicating a possible mechanism which involves HA in cell-to-cell and cell-to-matrix interactions (52, 53). In contrast to these reports, Mikami et al. showed that systemic inhibition of HA synthesis by oral 4-MU administration promotes the development of tumor in mice with liver tumors induced by administration of thioacetamide (TAA) (79). A possible explanation for this opposite result could be associated with the HCC model used by the authors. The administration of TAA induces DNA damage by increasing the levels of reactive oxygen species (ROS) and affecting the oxidative status of the liver microenvironment. Thus, HA inhibition at early stages could be affecting the documented protective action of HA during oxidative damage (80). At this time, 4-MU administration would be detrimental, perpetuating the damage of TAA and accelerating its carcinogenic action.

These results suggest that 4-MU administration could have a positive impact on the treatment of HCC by affecting angiogenic factors as well as hepatic CSCs. However, as commented in section 2.2, further preclinical studies will be required to adjust the moment of its application and the length of its use according to the tumor stage to avoid systemic alterations. On the other hand, analysis of the data about the interaction of 4-MU with other drugs are also necessary to determine whether they could affect its antitumoral action in HCC.

### 3.7 Bone-Derived Tumors

Osteosarcoma (OS), the most common primary bone tumor, is responsible for considerable morbidity and mortality due to its high rates of pulmonary metastasis. Although the prognosis of OS patients has improved dramatically with the introduction of chemotherapy, cases with metastases or an unresectable tumor still have a poor prognosis (54). Several researchers have suggested the involvement of a HA‐rich ECM in the tumorigenicity of OS cells, and proposed that suppression of this HA‐rich ECM leads to inhibition of malignant cell behavior (81–83). Arai et al. demonstrated that 4-MU reduces the formation of functional cell-associated matrices in OS cells and inhibits cell proliferation, migration, and invasion, resulting in the reduction of tumorigenicity and lung metastasis (54). These authors further studied 4-MU treatment in *in vivo* models of OS and found that, although it showed only a mild inhibitory effect on the growth of the primary tumor, it markedly inhibited the development of lung metastasis (54).

4-MU treatment has also shown antitumor effects on low‐grade chondrosarcoma, which is the second most common primary malignant bone tumor and a tumor generally considered resistant to conventional chemo‐ and radiotherapy (84). This type of tumor is characterized by the formation of a HA-rich ECM which has been proposed to be associated with drug resistance (85). Hamada et al. determined that, in chondrosarcoma cells, inhibition of HA synthesis by 4-MU suppressed cell proliferation, migration, and invasiveness, and that, *in vivo*, daily administration of 4-MU markedly inhibited local tumor growth and significantly suppressed the amount of HA in tumoral tissue (55).

Regarding fibrosarcoma, another of the most common bone-derived tumors, some reports have also shown a positive effect of 4-MU treatment on the sensitivity of cells to radiotherapy (56, 57, 86, 87). In primary solid tumors, external radiotherapy is generally effective and non-invasive and improves local control in the target region. However, although radiotherapy is an effective adjuvant treatment, metastasis and radiation resistance are associated with poor prognosis in patients (88). Saga et al. have shown that 4-MU administration in combination with exposure to 2-Gy ionizing radiation reduced HA production, cell invasion and the metastatic potential of fibrosarcoma cells *in vitro* (86), suggesting that 4-MU could be a radio-sensitizing molecule. Besides, in a later study, these same authors determined that the radio-sensitizing effect of 4-MU was not completely associated with its inhibitory effect on HA synthesis and that 4-MU improved the radiosensitivity of fibrosarcoma cells by suppressing inflammation (56). Specifically, they revealed that 4-MU increased the sensitivity of fibrosarcoma cells to X-ray radiation by inhibiting the production of the pro-inflammatory cytokines IL-1β, IL-6 (87), IL-1α, IL-36γ and IL-37 (56). Recently, the authors demonstrated that the radio-sensitizing effects of 4-MU are intrinsically related to the suppression of antioxidant activity through previously discovered anti-inflammatory effects (57).

Even more, in a model of metastatic breast cancer, Urakawa et al. determined that 4‐MU suppressed metastatic lesions of bone *in vivo* and inhibited the expansion of osteolytic lesions and intraosseous tumor growth in breast cancer xenograft models by inhibiting HA accumulation in tumor tissues (49).

These results suggest that, in bone-derived tumors, 4-MU could be a beneficial adjuvant during radiotherapy by inducing radio-sensitization of tumor cells as a consequence of HA synthesis inhibition as well as by an independent mechanism associated with the modulation of inflammatory and oxidative factors.

### 3.8 Melanoma

Melanoma is one of the three main types of skin cancer, being the most serious form. The prognosis of melanoma has historically been poor, with a median survival of less than 12 months, which can be ascribed to the aggressive nature of the disease and low response rates to conventional chemotherapy (89). In recent years, although major therapeutic advances have been made, resistance to these new therapies has also emerged (90). Thus, new treatment modalities are needed to improve the outcome, and 4-MU is one of the candidate molecules for use in new therapeutic strategies. In this sense, different studies have evaluated the potential role of 4-MU as a modulator of melanoma progression.

Kudo et al. demonstrated that 4-MU inhibits the formation of cell surface HA by B16F-10 melanoma cells and its release into the culture medium. These authors also showed that 4-MU had no significant cytotoxic effects on cell growth, but inhibited the adhesion and locomotion abilities of melanoma cells in a dose-dependent manner (58). Since adhesion and locomotion are involved in the early stages of metastasis, these results suggest that HA-rich matrices adjacent to melanoma cells provide a suitable environment for metastasis. In line with these findings, Yoshihara et al. evaluated the role of 4-MU in melanoma metastasis *in vivo*, by pre-treating melanoma cells with 4-MU before mouse inoculation, showing both decreased cell surface HA formation and suppression of metastasis after injection (59). These authors also demonstrated that oral administration of 4-MU to mice decreased liver HA content, which also contributed to a suppressed liver metastasis (59). Thus, in agreement with the data published by Kudo et al. (58), both cell surface HA of melanoma cells and recipient liver HA can promote liver metastasis of melanoma *in vivo* (59), strongly supporting 4-MU as a potential anti-metastatic agent in a highly malignant tumor as melanoma.

Another interesting study that reinforces the anti-invasive role of 4-MU was carried out by Edward et al. These authors showed that 4-MU inhibited tumor cell growth and the activation of stromal HA synthesis by melanoma cell-derived factors (91). Specifically, they demonstrated that 4-MU caused a dose-dependent growth inhibition of fibroblast and melanoma cells. The inhibition of cell growth was more pronounced when fibroblasts were stimulated with C8161 melanoma cell-conditioned medium (91). In addition, 4-MU reduced the level of HA in fibroblast-contracted collagen lattices, and inhibited both the growth of melanoma cells and invasion into the lattices (91). These results allow concluding that 4-MU has an anti-proliferative effect on the melanoma microenvironment, not only suppressing HA synthesis, but also inhibiting the induction of stromal HA accumulation and the proliferation of fibroblasts and melanoma cells.

Based on its growth-inhibitory activities against melanoma cells, Abildgaard et al. have recently proposed 4-MU as a new drug candidate for melanoma treatment and combination with chemotherapy (60). These authors showed that 4-MU affected cellular metabolism through inhibition of glycolysis and increased ROS production, suggesting the involvement of oxidative stress in the cellular response (60).

### 3.9 Glioblastoma

Glioblastoma (GBM) represents the most malignant and deadly brain tumor in adults (92). Despite invasive treatment strategies, involving a triad of surgery, radiation and chemotherapy, patients inevitably relapse due to resistance and invasion within the brain parenchyma and succumb within 15 months post-diagnosis (92). It is noteworthy that the ECM of malignant gliomas, like GBM, contains higher amounts of HA than normal brain tissue, indicating that HA could be instrumental for tumor adhesion and invasion (93, 94). It has been proposed that the aggressiveness of GBM depends on the co-expression of HAS and hyaluronidases (95). In this sense, based on the fact that 4-MU is a small molecule able to cross the blood brain barrier (96), Pibuel et al. proposed its use as an interesting therapeutic strategy to complement GBM treatment (65). These authors demonstrated that, in the GL26 murine GBM cell line, 4-MU diminished HA synthesis while increasing apoptosis and decreasing cell proliferation and migration (66). Yan et al. found that alterations in HA metabolism, by silencing HAS3 or by treating with 4-MU, inhibited glioma cell proliferation by affecting the autophagy flux (67). Although these new results are encouraging, more investigations are needed to understand the action and mechanism of 4-MU in GBM cells.

### 3.10 Chronic Myeloid Leukemias

Leukemia is the general name for cancer that involves blood-forming cells. Among them, Chronic myeloid leukemia (CML) is a type of cancer where the myeloid lineage is affected and comprises a group of myeloproliferative neoplasms. In 2020, approximately 15% of new cancer cases diagnosed in adults in the USA were leukemias (97). Most patients have typical cytogenetic alterations, the Philadelphia chromosome (Ph1), and the BCR/ABL rearrangement, the latter of which produces an abnormal tyrosine kinase and allows the specific treatment with inhibitors of this kinase. However, a group of patients can be Ph1-negative and have worse prognosis and shorter survival than Ph1-positive patients. This group thus needs special attention to find a successful therapy. Although there are different well-established therapeutic strategies to control CML progression (98), some studies have analyzed the potential role of 4-MU in CML. Ban et al. demonstrated that 4-MU is able to induce apoptosis in K562 CML cells by activating the intrinsic apoptosis pathway (61). These authors found that treatment with 4-MU leads to apoptosis in K562 cells through poly (-ADP-ribose) polymerase (PARP) cleavage and alteration of the mitochondrial membrane potential (61). Interestingly, they also observed that the addition of exogenous soluble HA protects K562 cells from 4-MU-induced apoptosis (61), which suggests that the pro-apoptotic effect of 4-MU demonstrated on CML cells is directly related to the inhibition of HA synthesis. In line with this study, the same research group later demonstrated the molecular mechanism by which 4-MU promotes apoptosis in CML cells (62). They showed that 4-MU treatment induced caspase-dependent apoptosis characterized by diminished HA synthesis, in correlation with increased phosphorylation of p38 and PARP cleavage (62). These authors also showed the pro-apoptotic effect of 4-MU *in vivo*, where treatment of tumor-bearing mice with 4-MU significantly reduced tumor growth through the induction of apoptosis (62). These results, together with those of other studies, determine the role of 4-MU as a molecule that favors the response of CML to chemotherapy (63, 64) and suggest that 4-MU is an excellent candidate for use in combination with conventional therapeutic strategies.

## 4 Effects of 4-MU on Specific Components of the Tumor Microenvironment

**Figure 1:**
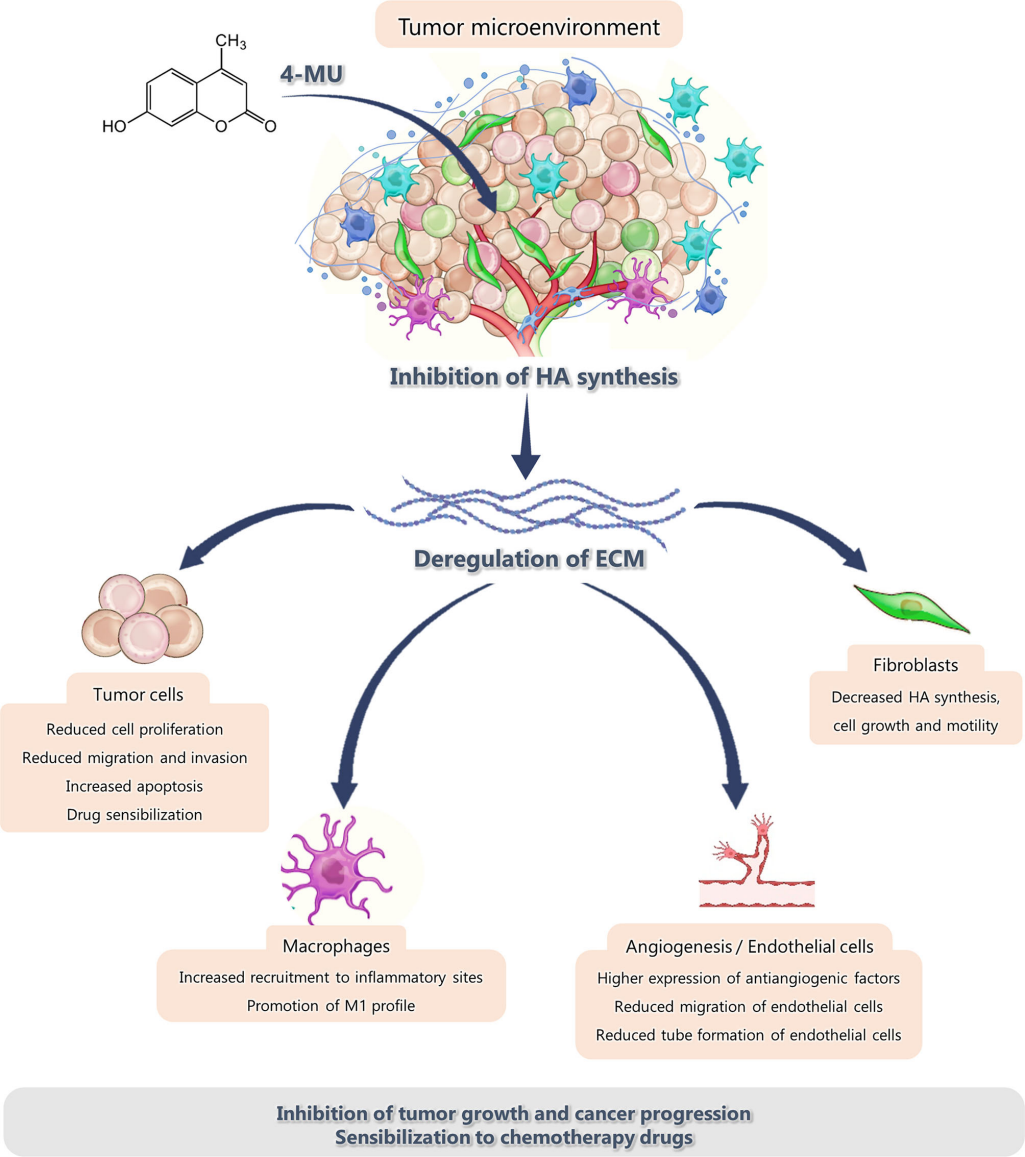
Effect of 4-MU treatment on the components of the tumor microenvironment.

Thanks to the numerous advances in the understanding of tumor biology and cancer progression, it is well known that the microenvironment where a tumor resides and develops is just as important and critical for its growth as tumor cells themselves. Therefore, it has been proposed that the modulation of the tumor microenvironment (TME) is particularly important to improve tumor response to cancer therapies (99). The TME is composed of non-cellular and cellular components. For decades, the specific role of non-cellular components of this microenvironment has been studied, focusing on the ECM components which can modulate tumor behavior. Even more, several functions of the different cell types associated with the tumor, such as immune cells, endothelial cells and mesenchymal stem cells, have been demonstrated. The modulation of the TME caused by 4-MU treatment is summarized in **Figure 1**.

### 4.1 Effects of 4-MU on Tumor-Associated Cells

#### 4.1.1 Tumor-Associated Fibroblasts

The cellular components of the TME include not only tumor cells themselves, but also cancer-associated fibroblasts (100, 101). Some authors have described that the interactions between tumor cells and associated stromal fibroblasts stimulate the synthesis of HA, which, as already mentioned, is present in large amounts in several types of tumor (102, 103). Recently, Cheng et al. showed that co-cultivation of PDAC cells and stromal fibroblasts increased HA production, resulting in a marked increase in the migration of PDAC cells (39).

Other authors have also shown that increased levels of HA in the tumor stroma are associated with poor prognosis (31, 104). In this sense, Urakawa et al. analyzed the effect of 4-MU on tumor stromal cells, particularly in a murine fibroblast cell line, and showed that 4-MU decreased HA levels, cell growth and motility of fibroblasts (49). Also, in a murine bone metastasis model of breast cancer, these authors showed that 4-MU administration decreased the accumulation of HA around both tumor and stromal cells, being well marked in the regions adjacent to bone which correspond to the stroma, where fibroblasts are generally abundant (49). In line with these results, Edward et al. showed that 4-MU inhibited fibroblast growth and reduced HA levels in fibroblast-contracted collagen lattices, which in turn inhibited both the growth and invasion by melanoma cell culture in this condition (91), indicating that the remodeling of the tumor stroma affects tumor development and metastatic capacity.

#### 4.1.2 Macrophages

Macrophages (MØ) are the main infiltrating immune cells of the TME. They differentiate from monocytes of systemic circulation in response to different stimuli from the environment and can exhibit two phenotypic profiles, M1 and M2. Despite these cells present high plasticity, MØ classically can be identified as M1 cells, that actively express HLA-DR and CD197 and have intrinsic phagocytosis capacity. Contrary, M2 cells express high levels of CD163, CD209, CD206 and CCL2 with anti-inflammatory functions (105). Specially, tumor-associated macrophages (TAMs) can be considered as M2-like phenotype due to anti-inflammatory cytokines of the TME. They can induce angiogenesis and lymphangiogenesis, by the release of growth factors like VEGF, FGF, PDGF and TGF-β and matrix-remodeling proteases. Moreover, they can suppress adaptive and innate immune responses by the release of anti-inflammatory factors like IL-10, TGF-β, and PD1L (106). Therefore, TAMs promote the growth and spread of tumor cells and reduce patient’s survival. Because of this, TAMs have been proposed as therapeutic targets for cancer therapy. Additionally, HA from the tumor ECM can modulate MØ adhesion, migration and activation through its surface receptors, depending on the size of the molecule. It is well known that low-molecular-weight HA stimulates the expression of inflammatory cytokines and chemokines and growth factors (107). The interaction between receptors such as CD44 and TLR and HA fragments induces the expression of inflammatory mediators in murine and human macrophages (108, 109) and can act as a danger signal by promoting antigen-specific T-cell response (110). On the other hand, high-molecular-weight HA has anti-inflammatory and antiproliferative properties, like regulatory T-cell activation (111, 112). At our lab, in a breast cancer model, we have previously demonstrated that high-molecular-weight HA promotes MØ pro-angiogenic capabilities (113). For this reason, HA-inhibitors like 4-MU could be a promising therapy. However, the effect of 4-MU on immune cells in the context of cancer is poorly studied. In an atherosclerosis *in vivo* model, Nagy et al. showed that 4-MU oral administration in mice led to a significant increase in MØ recruitment in atherosclerotic lesions, promoting an inflammatory response and the development of the disease (26). In addition, Rodríguez et al. demonstrated that long-term 4-MU oral administration in mice with hepatocarcinoma caused, in MØ, an increase in the secretion of pro-inflammatory cytokines, IL-1β and TNF-α, and a decrease in anti-inflammatory cytokines, IL-10 and TGF-β, indicating the polarization of these cells towards an M1 profile in tumor and non-tumor regions. These examples demonstrate that 4-MU action over immune cells is context-dependent.

#### 4.1.3 Endothelial Cells

Endothelial cells are involved in angiogenesis, i.e. the formation of new blood vessels by sprouting from preexisting vessels. In tumors, this process is essential as it allows their growth and dissemination. Although this process is targeted by different therapeutic drugs approved for use in cancer, development of resistance has been observed. Thus, since 4-MU can affect endothelial cell behavior, it could be a good strategy to maximize anti-angiogenic therapy (114). Garcia-Vilas et al. have shown evidence of action of 4-MU over endothelial cells. These authors observed that 4-MU inhibited cell growth, was able to generate new vessels without affecting the migration capacity, and enhanced the expression of metalloproteinases (25). Finally, by using different angiogenesis models *in vivo*, they observed that 4-MU led to a significant reduction of this process (25). In an HCC model, Piccioni et al. found evidence of 4-MU effect on endothelial cells in the TME (27). They observed that 4MU-treated mice showed significantly diminished systemic levels of VEGF and expression of the specific vascular marker CD31. They also found that 4-MU was able to inhibit endothelial cell migration and tube formation, demonstrating that 4-MU has an anti-angiogenic activity in HCC (27). Similar results have been observed in a model of prostate cancer (44). However, since little is known about the direct action of 4-MU over endothelial cells in cancer, this topic should be further explored.

### 4.2 Effects of 4-MU Treatment on the Non-Cellular TME

The ECM is the non-cellular component of the TME. During embryonic development and organ homeostasis, the composition of the ECM is tightly regulated. However, in diseases such as cancer, it is usually deregulated and disorganized, and undergoes extensive remodeling, acting as a key player driving disease progression (76, 115). In this sense, extremely high interstitial fluid pressures and a dense ECM combine to limit the delivery and distribution of therapeutic agents in solid tumors (116). In addition, high concentrations of HA cause an expansion of the ECM, which contributes to increased tumor interstitial pressure, which retards the delivery and distribution of drugs from the vessels into the tumor (117–120). Therefore, strategies to remove HA or block its synthesis may improve drug delivery into solid tumors. In this sense, several studies have shown that the inhibition of HA synthesis by enzymatic agents, like PEGylated recombinant hyaluronidase (PEGPH20), normalize interstitial fluid pressure and re-expand the microvasculature, improving the delivery, distribution and accumulation of drugs in tumors (117–119). Regarding this, Dufort et al. showed that the systemic treatment of mice with PEGPH20 reduced the extracellular levels of HA and interstitial pressure, thus removing a significant barrier for drug delivery in PDAC (117). Other authors also showed that the treatment with PEGH20 *in vivo* reduces HA content, induces the re-expansion of the microvasculature, and consequently improves gemcitabine and DOX uptake in murine PDAC (118, 119). This example demonstrates the potential of targeting the ECM/stroma and modulating the mechanical properties of the surrounding microenvironment, as an anti-PDAC therapy.

Unfortunately, recent research has shown that the promising results obtained for PEGH20 in a phase I/II clinical trial in PDAC (121) did not translate into the subsequent phase III study HALO 301 (122) and further development of this drug was stopped. This highlights the importance of looking for other strategies that allow blocking HA synthesis. In this context, the use of 4-MU may be a promising strategy. An interesting research has shown that 4-MU significantly reduced the amount of tumor HA, leading to a significant decrease in tumor interstitial pressure and achieving improved tumor perfusion in murine colorectal carcinoma (36). Similarly, as described above, in a model of pancreas tumor, 4-MU was able to remodel the ECM-generated interstitial gap within the tumor cell by inhibiting HA production (72).

However, it is likely that 4-MU can also affect the synthesis and organization of other ECM components, such as other non-cellular components of the TME. In this regards, Keller et al. found that 4-MU reduced both versican and fibronectin in trabecular meshwork cells of the eye (123). Even more, Andreichenko et al. confirmed that 4-MU inhibits ECM deposition by directly affecting the production not only of HA, but also of Col1a, a major form of collagens contributing to ECM remodeling in liver fibrosis (124). It was observed that other glycosaminoglycans, such as chondroitin and heparin sulfates, were sensitive to 4-MU treatment in epidermal keratinocyte cultures. In this sense, a 4-MU concentration-dependent decrease was found in the production of these glycosaminoglycans, although the effect was greater on HA In epidermal keratinocyte cultures, Rilla et al. observed that other glycosaminoglycans, such as chondroitin and heparin sulfates, were sensitive to 4-MU treatment. They found that the production of these glycosaminoglycans decreased in a 4-MU concentration-dependent manner, although the effect was greater on HA (125). In addition, an effect of 4-MU on matrix metalloproteinases (MMPs), a family of proteolytic enzymes that degrade many ECM components and play an important role in tissue degradation and remodeling under various physiological and pathological conditions, has been observed. Nakamura et al. reported that, in human skin fibroblasts, 4-MU induces MMP2 activation (126). Surprisingly, in pathological conditions, 4-MU shows a differential effect. Nakamura et al. reported that, in a human lymphoma cell line as well as in other cultured human carcinoma cells, 4-MU inhibited MMP9, an inhibition that could not be mimicked by treatment of the cells with hyaluronidase (127). These studies show that 4-MU may target ECM components other than HA. Even more, as described above, in a model of fibrosarcoma cells, 4-MU was able to remodel the surrounding TME by inhibiting the production of pro-inflammatory cytokines, altering other non-cellular components of the TME, different from the ECM (56, 87).

Although many reports have highlighted the importance of 4-MU in inhibiting HA synthesis, it could also be affecting the synthesis of other ECM components like proteoglycans and have biological effects on soluble tumoral factors. In fact, further studies about its effect on other non-cellular components of the TME, their interaction, and their role in cancer pathogenesis will be necessary. For example, it will be interesting to investigate the impact of 4-MU modulation over different ECM components and the mechanical properties of the surrounding TME.

## 5 4-MU Treatment as a New Strategy of Co-Adjuvant Drug on Conventional Antineoplastic Therapies

One of the most important challenges of antineoplastic therapies is to adjust the treatment to the needs of each patient and reduce the toxicity caused by conventional antitumoral strategies. Several scientific studies have reported the key role of the pericellular HA-rich ECM as a biological barrier in the TME. Among the processes controlled by this natural barrier are the modulation of immune effectors (35, 113), the inhibition of diffusion of chemotherapeutic drugs (128) and the difficult uptake of DNA transgene complexes in gene therapy (129). Furthermore, previous studies from our laboratory and other authors have shown that ECM components play important roles in acquired resistance to anticancer drugs (34, 130, 131). Therefore, the development of novel cancer treatments that target HA by altering the ECM represents a pioneering approach to the treatment of several cancers.

According to the evidence collected so far, 4-MU represents one of the candidate molecules for drug repositioning in cancer therapy. While the potential advantage of 4-MU as an adjunct in cancer therapy could improve therapeutic efficacy and reduce toxicities, the greatest challenge is the lack of strong scientific evidence to support its approval. Therefore, crucial human clinical studies have yet to be performed to respond to this need. Nevertheless, numerous scientific reports in the early stages of research have studied the role of 4-MU as a co-adjuvant of conventional antineoplastic treatments. Since it has been previously demonstrated that 4-MU mediates the inhibition of HA synthesis and pericellular HA matrix formation, this molecule would increase the efficacy of anticancer treatments.

In a study of alternative therapies applicable to pancreatic cancer, Nakazawa et al. showed that pre-treatment of KP1-NL cells with 4-MU increased the anticancer effect of gemcitabine (40). Particularly, these authors showed that pancreatic cancer cells are enclosed by HA-rich coats, and that 4-MU treatment inhibited the formation of HA pericellular coat, which promoted the perfusion and uptake of gemcitabine (41). These results were also confirmed in an *in vivo* murine model, where co-administration of 4-MU and gemcitabine to tumor-bearing mice reduced the size of the primary and metastatic tumors (40). These data suggest that the combination of 4-MU and gemcitabine is effective against human pancreatic cancer cells and tumor progression *in vivo*. Regarding the possible use of 4-MU as a modulator of chemotherapy in pancreatic cancer, Yoshida et al. found a similar effect in combination with 5-fluorouracil (5-FU) (42). These authors showed that 4-MU administration changed the antitumor efficacy of 5-FU, enhancing its cytotoxicity *in vitro* and *in vivo* and that combined treatments of 5-FU and 4-MU inhibited cell proliferation and enhanced the intracellular concentration of 5-FU *in vitro* (42). In the *in vivo* model, the authors found that mouse tumors treated with 5-FU and 4-MU decreased in size and animal survival was prolonged, in addition to a decrease in the cohesiveness of the intercellular space, which favored 5-FU perfusion and activity (42).

These findings are consistent with a recent study showing that chemotherapy with carboplatin (CBP) induces HA synthesis, which can contribute to chemoresistance by regulating ABC transporter expression in ovarian cancer (132). Specifically, this study determined that, in combination with CBP, 4-MU treatment significantly decreased ovarian cancer cell survival and increased apoptosis compared to CBP alone (132). In addition, this combined treatment reduced the expression of cancer stem cell markers such as ALDH1 and ABCG2 (132). Furthermore, 4-MU inhibits the invasion ability of chemoresistant primary cells *in vivo*, demonstrating that HA inhibition is a promising new strategy to overcome chemoresistance and improve ovarian cancer survival (132).

The effect of 4-MU as a promoter of chemotherapeutic treatment has also been determined in other types of tumors such as glioblastoma, the most frequent primary tumor of the central nervous system (133). In this work, the potential antitumor effect of 4-MU was tested in combination with temozolomide on GL26 glioblastoma cells. As expected, 4-MU decreased HA synthesis, but also diminished cell proliferation and induced apoptosis while reducing cell migration and the activity of MMPs. Besides, 4-MU sensitized GL26 cells to the effect of temozolomide and showed selective toxicity in tumor cells without exhibiting neurotoxic effects, highlighting its potential usefulness to improve glioblastoma treatment (66).

Another antineoplastic strategy mainly used for cancer treatment is radiotherapy. In this regards, 4-MU has been proposed as a positive modulator of radiotherapy response in fibrosarcoma. Saga et al. reported that co-administration of 4-MU enhanced the lethality of X-ray irradiation in HT1080 human fibrosarcoma cells and decreased their invasiveness (86). After that, the authors continued investigating the molecular bases of their discovery and found that co-administration of 4-MU suppressed the activation of IL-6 and IL-8 after X-ray irradiation (86). Similar results have been observed for the upstream signaling component IL-1 (87). These results indicate that the radiosensitivity of fibrosarcoma cells is improved by suppressing inflammation through the administration of 4-MU.

Consistent results have also been found when evaluating 4-MU as a co-adjuvant of antineoplastic therapies against melanoma and CML. In the case of melanoma, one of the therapeutic strategies is based on the inhibition of the BRAF oncogene, since the most prevalent BRAF mutation in melanoma is directly associated with cellular metabolic reprogramming by the Warburg effect (134, 135). Therefore, treatment with BRAF inhibitors reverses the Warburg effect and stimulates mitochondrial activity, which favors disease control (136, 137). In this regards, Abildgaard et al. demonstrated that 4-MU potentiates the antitumor effect of the BRAF inhibitor vemurafenib (60). Particularly, they found that the combination of 4-MU and vemurafenib was more effective in reducing viability of ED-013 and ED-196 melanoma cells than vemurafenib treatment alone, inducing cell cycle arrest in G1 phase. These authors also found that 4-MU plus vemurafenib treatment increased the cellular production of ROS (60).

Similarly, different studies have proposed 4-MU as a candidate molecule for co-adjuvant treatments for CML. Uchakina et al. showed that 4-MU sensitizes K562 cells to doxorubicin treatment, by inhibiting HA synthesis and increasing apoptosis rates through p38 activation and PARP cleavage (63). Lompardía et al. found similar results when combining 4-MU treatment with the chemotherapeutic agent vincristine on K562 and K562 vincristine-resistant cells (Kv562) (64). These authors revealed that 4-MU decreased tumor cell proliferation and sensitized Kv562 resistant cells to vincristine effect and determined that 4-MU effect was related to the inhibition of P-glycoprotein and the induction of senescence (64). These results support the potential use of 4-MU for combination of therapies in cancer and may encourage preclinical validation and clinical testing of such treatment strategies.

## 6 4-MU Repurposed From a Dietary Component to an Anticancer Drug: Potentials for Its Repositioning

By definition, “drug repositioning” is a method that can help the conventional drug discovery process by using existing drugs for treatment of a different disease instead of their original indication (138). During the COVID-19 pandemic, it has been shown that this reasoning about the reuse of drugs is an effective and fast way to provide a treatment solution in a short time (139). The integration of bioinformatics data tools or “Big Data” (-omic data, sequencing DNA/RNA, molecular modeling, tumor biobanks, clinical trials, etc.) and experimental data offers the possibility to identify how feasible drugs are to be reused (138). 4-MU, originally identified as a hepatoprotective component, could be considered for this purpose and be now used as an antitumoral drug. The results described in this review suggest that this drug could be a good option to improve efficacy and reduce toxicity of current cancer treatment.

## 7 Conclusions and Perspectives

The mechanisms of action of 4-MU are not yet known in detail. However, different results suggest that some of these mechanisms may be independent of HA synthesis inhibition. In this sense, over the last years, some authors have described HA-independent effects for 4-MU toxicity (13, 75). For example, in trabecular meshwork cells of the eye, Keller et al. found that 4-MU reduced the ECM components versican and fibronectin, and that the addition of exogenous HA failed to reverse the effects of 4-MU (123). Since versican and fibronectin can affect tumor progression and development (140), it is likely that 4-MU can also affect the synthesis and organization of other ECM components to mediate its effects in tumor cells. However, more studies are required to corroborate this hypothesis. Together, these reports reinforce that 4-MU may have different anti-tumor mechanisms depending on the type of cancer. However, toxicological, pharmacokinetic and pharmacodynamic aspects that determine the treatment regimen (way of administration, doses that impact on its bioavailability, time of interval between them and schedule) should be extensively reviewed in preclinical studies. An important study performed by Kuipers et al. in an EAE mouse model determined that, to observe a systemic decrease in HA levels, 4-MU should be administered for 7 days or more and that longer use does not completely reduce HA levels (141). Besides, they observed that, after oral administration, 4-MU is rapidly metabolized to 4-MUG and in minor proportion to 4-MUS and, since there is a low bioavailability of 4-MU, high doses are required to reach a considerable percentage at systemic level (141). Thus, its metabolites and bioavailability are important points to be considered in the use of 4-MU without risk of toxic effect. In fact, Nagy et al. showed that 4-MUG is a bioactive metabolite that can be hydrolyzed into 4-MU and that 4-MUG also had effects similar to those of 4-MU *in vivo* (142), suggesting that studies using 4-MU should rethink the concept of its bioavailability.

All these reports suggest the feasibility of using 4-MU in cancer treatment. However, deepening the knowledge of its mechanisms of action and other pharmacological aspects will allow its application in clinical trials and its consideration as a therapeutic option, in combination or not, in current oncology treatments.

## Author Contributions

DV contributed to the design of the review and combination of subtopics. DV, AI, PR, FS, IS, and LA each wrote the subtopic of the review. LA contributed to the conception and design of the review. DV, IS, and LA contributed to the final manuscript revision. All authors contributed to the article and approved the submitted version.

## Funding

UNNOBA, SIB 0561/2019, UNNOBA (to LA and IS), PICTO UNNOBA 2019-00011 (to LA).

## Conflict of Interest

The authors declare that the research was conducted in the absence of any commercial or financial relationships that could be construed as a potential conflict of interest.

## Publisher’s Note

All claims expressed in this article are solely those of the authors and do not necessarily represent those of their affiliated organizations, or those of the publisher, the editors and the reviewers. Any product that may be evaluated in this article, or claim that may be made by its manufacturer, is not guaranteed or endorsed by the publisher.

## References

[1] Newman DJ Cragg GM. Natural Products as Sources of New Drugs Over the Nearly Four Decades From 01/1981 to 09/2019. J Natural Products (2020) 83:770–803. doi: 10.1021/acs.jnatprod.9b01285 32162523

[2] Jang DS Park EJ Hawthorne ME Vigo JS Graham JG Cabieses F. Potential Cancer Chemopreventive Constituents of the Seeds of Dipteryx Odorata (Tonka Bean). J Nat Prod (2003) 66(5):583–7. doi: 10.1021/np020522n 12762787

[3] Lacy A. Studies on Coumarins and Coumarin-Related Compounds to Determine Their Therapeutic Role in the Treatment of Cancer. Curr Pharm Des (2005) 10(30):3797–811. doi: 10.2174/1381612043382693 15579072

[4] Arshad A Osman H Bagley MC Lam CK Mohamad S Zahariluddin ASM. Synthesis and Antimicrobial Properties of Some New Thiazolyl Coumarin Derivatives. Eur J Med Chem (2011) 46(9):3788–94. doi: 10.1016/j.ejmech.2011.05.044 21712145

[5] Abdelhafez OM Amin KM Batran RZ Maher TJ Nada SA Sethumadhavan S. Synthesis, Anticoagulant and PIVKA-II Induced by New 4-Hydroxycoumarin Derivatives. Bioorg Med Chem (2010) 18(10):3371–8. doi: 10.1016/j.bmc.2010.04.009 20435480

[6] Xia Y Yang ZY Xia P Hackl T Hamel E Mauger A. Antitumor Agents. 211. Fluorinated 2-Phenyl-4-Quinolone Derivatives as Antimitotic Antitumor Agents. J Med Chem (2001) 44(23):3932–6. doi: 10.1021/jm0101085. 11689079

[7] Sunthitikawinsakul A Kongkathip N Kongkathip B Phonnakhu S Daly JW Spande TF. Coumarins and Carbazoles From Clausena Excavata Exhibited Antimycobacterial and Antifungal Activities. Planta Med (2003) 69(2):155–7. doi: 10.1055/s-2003-37716. 12624822

[8] Bhattacharyya SS Paul S Mandal SK Banerjee A Boujedaini N Khuda-Bukhsh AR. A Synthetic Coumarin (4-Methyl-7 Hydroxy Coumarin) has Anti-Cancer Potentials Against DMBA-Induced Skin Cancer in Mice. Eur J Pharmacol (2009) 614(1–3):128–36. doi: 10.1016/j.ejphar.2009.04.015. 19393233

[9] Fylaktakidou K Hadjipavlou-Litina D Litinas K Nicolaides D. Natural and Synthetic Coumarin Derivatives With Anti-Inflammatory / Antioxidant Activities. Curr Pharm Des (2005) 10(30):3813–33. doi: 10.2174/1381612043382710. 15579073

[10] Mazimba O. Umbelliferone: Sources, Chemistry and Bioactivities Review. Bull Fac Pharmacy Cairo Univ (2017) 55(2):223–32. doi: 10.1016/j.bfopcu.2017.05.001

[11] Kultti A Pasonen-Seppänen S Jauhiainen M Rilla KJ Kärnä R Pyöriä E. 4-Methylumbelliferone Inhibits Hyaluronan Synthesis by Depletion of Cellular UDP-Glucuronic Acid and Downregulation of Hyaluronan Synthase 2 and 3. Exp Cell Res (2009) 315(11):1914–23. doi: 10.1016/j.yexcr.2009.03.002 19285976

[12] Spinelli FM Vitale DL Sevic I Alaniz L. Hyaluronan in the Tumor Microenvironment. Adv Exp Med Biol (2020) 1245:67–83. doi: 10.1007/978-3-030-40146-7_3 32266653

[13] Ishizuka S Askew EB Ishizuka N Knudson CB Knudson W. 4-Methylumbelliferone Diminishes Catabolically Activated Articular Chondrocytes and Cartilage Explants *via* a Mechanism Independent of Hyaluronan Inhibition. J Biol Chem (2016) 291(23):12087–104. doi: 10.1074/jbc.M115.709683 PMC4933260 27129266

[14] Rodríguez MM Onorato A Cantero MJ Domínguez L Bayo J Fiore E. 4-Methylumbelliferone-Mediated Polarization of M1 Macrophages Correlate With Decreased Hepatocellular Carcinoma Aggressiveness in Mice. Sci Rep (2021) 11(1):6310. doi: 10.1038/s41598-021-85491-0 33737571 PMC7973733

[15] Bhagavan NV Ha C-E. Chapter 14 - Carbohydrate Metabolism II: Gluconeogenesis, Glycogen Synthesis and Breakdown, and Alternative Pathways. In: Second E, Editor. Bhagavan N V, Ha C-EBT-E of MB. San Diego: Academic Press (2015). p. 205–25.

[16] Egan D O’kennedy R Moran E Cox D Prosser E Thornes RD. The Pharmacology, Metabolism, Analysis, and Applications of Coumarin and Coumarin-Related Compounds. Drug Metab Rev (1990) 22(5):503–29. doi: 10.3109/03602539008991449 2078993

[17] Ritschel WA Hoffmann KA. Pilot Study on Bioavailability of Coumarin and 7-Hydroxycoumarin Upon Peroral Administration of Coumarin in a Sustained-Release Dosage Form. J Clin Pharmacol (1981) 21(7):294–300. doi: 10.1002/j.1552-4604.1981.tb01770.x 7263928

[18] Vassallo JD Hicks SM Born SL Daston GP. Roles for Epoxidation and Detoxification of Coumarin in Determining Species Differences in Clara Cell Toxicity. Toxicol Sci (2004) 82(1):26–33. doi: 10.1093/toxsci/kfh237 15282406

[19] Marshall ME Mohler JL Edmonds K Williams B Butler K Ryles M. An Updated Review of the Clinical Development of Coumarin (1,2-Benzopyrone) and 7-Hydroxycoumarin. J Cancer Res Clin Oncol (1994) 120(1 Supplement):S39–42. doi: 10.1007/BF01377124 PMC12200304 8132703

[20] Pelkonen O Raunio H Rautio A Pasanen M LM. Coumarins: Biology, Applications and Mode of Action. (1997). https://oehha.ca.gov/media/downloads/crnr/coumarinhid.pdf (Accesed May 10, 2021)

[21] Lake BG. Coumarin Metabolism, Toxicity and Carcinogenicity: Relevance for Human Risk Assessment. Food Chem Toxicol (1999) 37:423–53. doi: 10.1016/S0278-6915(99)00010-1 10418958

[22] Menezes JCJMDS Diederich M. Translational Role of Natural Coumarins and Their Derivatives as Anticancer Agents. Future Med Chem (2019) 11(9):1057–82. doi: 10.4155/fmc-2018-0375 31140865

[23] Lake BG Evans JG Chapuis F Walters DG Price RJ. Studies on the Disposition, Metabolism and Hepatotoxicity of Coumarin in the Rat and Syrian Hamster. Food Chem Toxicol (2002) 40(6):809–23. doi: 10.1016/S0278-6915(02)00036-4 11983276

[24] Krawzak HW Heistermann HP Andrejewski K Hohlbach G. Postprandial Bile-Duct Kinetics Under the Influence of 4-Methylumbelliferone (Hymecromone). Int J Clin Pharmacol Ther (1995) 33(10):569–72. 8574509

[25] García-Vilas JA Quesada AR Medina MÁ. 4-Methylumbelliferone Inhibits Angiogenesis *In Vitro* and *In Vivo* . J Agric Food Chem (2013) 61(17):4063–71. doi: 10.1021/jf303062h 23581646

[26] Nagy N Freudenberger T Melchior-Becker A Röck K Ter Braak M Jastrow H. Inhibition of Hyaluronan Synthesis Accelerates Murine Atherosclerosis: Novel Insights Into the Role of Hyaluronan Synthesis. Circulation (2010) 122(22):2313–22. doi: 10.1161/CIRCULATIONAHA.110.972653 21098434

[27] Piccioni F Fiore E Bayo J Atorrasagasti C Peixoto E Rizzo M. 4-Methylumbelliferone Inhibits Hepatocellular Carcinoma Growth by Decreasing IL-6 Production and Angiogenesis. Glycobiology (2015) 25(8):825–35. doi: 10.1093/glycob/cwv023 25882295

[28] Olivares CN Alaniz LD Menger MD Barañao RI Laschke MW Meresman GF. Inhibition of Hyaluronic Acid Synthesis Suppresses Angiogenesis in Developing Endometriotic Lesions. PloS One (2016) 11(3):e0152302. doi: 10.1371/journal.pone.0152302 27018976 PMC4809563

[29] Nagy N Kuipers HF Frymoyer AR Ishak HD Bollyky JB Wight TN. 4-Methylumbelliferone Treatment and Hyaluronan Inhibition as a Therapeutic Strategy in Inflammation, Autoimmunity, and Cancer. Front Immunol (2015) 6(MAR):1–11. doi: 10.3389/fimmu.2015.00123 25852691 PMC4369655

[30] Theocharis AD Tsara ME Papageorgacopoulou N Karavias DD Theocharis DA. Pancreatic Carcinoma Is Characterized by Elevated Content of Hyaluronan and Chondroitin Sulfate With Altered Disaccharide Composition. Biochim Biophys Acta - Mol Basis Dis (2000) 1502(2):201–6. doi: 10.1016/S0925-4439(00)00051-X 11040445

[31] Lipponen P Aaltomaa S Tammi R Tammi M Ågren U Kosma VM. High Stromal Hyaluronan Level is Associated With Poor Differentiation and Metastasis in Prostate Cancer. Eur J Cancer (2001) 37(7):849–56. doi: 10.1016/S0959-8049(00)00448-2 11313172

[32] Sironen RK Tammi M Tammi R Auvinen PK Anttila M Kosma VM. Hyaluronan in Human Malignancies. Exp Cell Res (2011) 17(4):383–91. doi: 10.1016/j.yexcr.2010.11.017 21134368

[33] Toole BP. Hyaluronan-CD44 Interactions in Cancer: Paradoxes and Possibilities. Clin Cancer Res (2009) 15(24):7462–68. doi: 10.1158/1078-0432.CCR-09-0479 PMC2796593 20008845

[34] Vitale DL Spinelli FM Del Dago D Icardi A Demarchi G Caon I. Co-Treatment of Tumor Cells With Hyaluronan Plus Doxorubicin Affects Endothelial Cell Behavior Independently of VEGF Expression. Oncotarget (2018) 9(93):36585–602. doi: 10.18632/oncotarget.26379 PMC6290962 30564299

[35] McBride WH Bard JBL. Hyaluronidase-Sensitive Halos Around Adherent Cells: Their Role in Blocking Lymphocyte-Mediated Cytolysis. J Exp Med (1979) 149(2):507–15. doi: 10.1084/jem.149.2.507 PMC2184811 762499

[36] Malvicini M Fiore E Ghiaccio V Piccioni F Rizzo M Olmedo Bonadeo L. Tumor Microenvironment Remodeling by 4-Methylumbelliferone Boosts the Antitumor Effect of Combined Immunotherapy in Murine Colorectal Carcinoma. Mol Ther (2015) 23(9):1444–55. doi: 10.1038/mt.2015.112 PMC4817882 26105158

[37] Nagase H Kudo D Suto A Yoshida E Suto S Negishi M. 4-Methylumbelliferone Suppresses Hyaluronan Synthesis and Tumor Progression in SCID Mice Intra-Abdominally Inoculated With Pancreatic Cancer Cells. Pancreas (2017) 46(2):190–7. doi: 10.1097/MPA.0000000000000741 PMC5266424 27846148

[38] Sato N Cheng XB Kohi S Koga A Hirata K. Targeting Hyaluronan for the Treatment of Pancreatic Ductal Adenocarcinoma. Acta Pharm Sin B (2016) 6(2):101–5. doi: 10.1016/j.apsb.2016.01.002 PMC4788704 27006892

[39] Cheng XB Sato N Kohi S Koga A Hirata K. 4-Methylumbelliferone Inhibits Enhanced Hyaluronan Synthesis and Cell Migration in Pancreatic Cancer Cells in Response to Tumor-Stromal Interactions. Oncol Lett (2018) 15(5):6297–301. doi: 10.3892/ol.2018.8147 PMC5920249 29725394

[40] Nakazawa H Yoshihara S Kudo D Morohashi H Kakizaki I Kon A. 4-Methylumbelliferone, a Hyaluronan Synthase Suppressor, Enhances the Anticancer Activity of Gemcitabine in Human Pancreatic Cancer Cells. Cancer Chemother Pharmacol (2006) 57(2):165–70. doi: 10.1007/s00280-005-0016-5 16341905

[41] Yoshida E Kudo D Nagase H Shimoda H Suto S Negishi M. Antitumor Effects of the Hyaluronan Inhibitor 4-Methylumbelliferone on Pancreatic Cancer. Oncol Lett (2016) 12(4):2337–44. doi: 10.3892/ol.2016.4930 PMC5038477 27698797

[42] Yoshida E Kudo D Nagase H Suto A Shimoda H Suto S. 4-Methylumbelliferone Decreases the Hyaluronan-Rich Extracellular Matrix and Increases the Effectiveness of 5-Fluorouracil. Anticancer Res (2018) 38(10):5799–804. doi: 10.21873/anticanres.12919 30275202

[43] Lokeshwar VB Lopez LE Munoz D Chi A Shirodkar SP Lokeshwar SD. Antitumor Activity of Hyaluronic Acid Synthesis Inhibitor 4-Methylumbelliferone in Prostate Cancer Cells. Cancer Res (2010) 70(7):2613–23. doi: 10.1158/0008-5472.CAN-09-3185 PMC2848908 20332231

[44] Yates TJ Lopez LE Lokeshwar SD Ortiz N Kallifatidis G Jordan A. Dietary Supplement 4-Methylumbelliferone: An Effective Chemopreventive and Therapeutic Agent for Prostate Cancer. J Natl Cancer Inst (2015) 107(7):1–10. doi: 10.1093/jnci/djv085 PMC4554252 25868577

[45] An G Park S Lee M Lim W Song G. Antiproliferative Effect of 4-Methylumbelliferone in Epithelial Ovarian Cancer Cells Is Mediated by Disruption of Intracellular Homeostasis and Regulation of PI3K/AKT and Mapk Signaling. Pharmaceutics (2020) 12(7):1–14. doi: 10.3390/pharmaceutics12070640 PMC7408106 32645961

[46] Anttila MA Tammi RH Tammi MI Syrjänen KJ Saarikoski SV Kosma VM. High Levels of Stromal Hyaluronan Predict Poor Disease Outcome in Epithelial Ovarian Cancer. Cancer Res (2000) 60(1). 10646867

[47] Tamura R Yokoyama Y Yoshida H Imaizumi T Mizunuma H. 4-Methylumbelliferone Inhibits Ovarian Cancer Growth by Suppressing Thymidine Phosphorylase Expression. J Ovarian Res (2014) 7(1):1–8. doi: 10.1186/s13048-014-0094-2 25304388 PMC4198731

[48] Wang R Zhou W Wang J Liu Y Chen Y Jiang S. Role of Hyaluronan and Glucose on 4-Methylumbelliferoneinhibited Cell Proliferation in Breast Carcinoma Cells. Anticancer Res (2015) 35(9):4799–806. 26254370

[49] Urakawa H Nishida Y Wasa J Arai E Zhuo L Kimata K. Inhibition of Hyaluronan Synthesis in Breast Cancer Cells by 4-Methylumbelliferone Suppresses Tumorigenicity *In Vitro* and Metastatic Lesions of Bone *In Vivo* . Int J Cancer (2012) 130(2):454–66. doi: 10.1002/ijc.26014 21387290

[50] Brett M-E Bomberger HE Doak GR Price MA McCarthy JB Wood DK. *In Vitro* Elucidation of the Role of Pericellular Matrix in Metastatic Extravasation and Invasion of Breast Carcinoma Cells. Integr Biol (2018) 10(4):242–52. doi: 10.1039/C7IB00173H PMC6556113 29623978

[51] Okuda H Kobayashi A Xia B Watabe M Pai SK Hirota S. Hyaluronan Synthase HAS2 Promotes Tumor Progression in Bone by Stimulating the Interaction of Breast Cancer Stem-Like Cells With Macrophages and Stromal Cells. Cancer Res (2012) 72(1):537–47. doi: 10.1158/0008-5472.CAN-11-1678 PMC3404816 22113945

[52] Sukowati CHC Anfuso B Fiore E Ie SI Raseni A Vascotto F. Hyaluronic Acid Inhibition by 4-Methylumbelliferone Reduces the Expression of Cancer Stem Cells Markers During Hepatocarcinogenesis. Sci Rep (2019) 9(1):1–11. doi: 10.1038/s41598-019-40436-6 30858465 PMC6411988

[53] Rodríguez MM Fiore E Bayo J Atorrasagasti C García M Onorato A. 4Mu Decreases CD47 Expression on Hepatic Cancer Stem Cells and Primes a Potent Antitumor T Cell Response Induced by Interleukin-12. Mol Ther (2018) 26(12):2738–50. doi: 10.1016/j.ymthe.2018.09.012 PMC6277513 30301668

[54] Arai E Nishida Y Wasa J Urakawa H Zhuo L Kimata K. Inhibition of Hyaluronan Retention by 4-Methylumbelliferone Suppresses Osteosarcoma Cells *In Vitro* and Lung Metastasis *In Vivo* . Br J Cancer (2011) 105(12):1839–49. doi: 10.1038/bjc.2011.459 PMC3251882 22045192

[55] Hamada S Nishida Y Zhuo L Shinomura T Ikuta K Arai E. Suppression of Hyaluronan Synthesis Attenuates the Tumorigenicity of Low-Grade Chondrosarcoma. J Orthop Res (2018) 36(6):1573–80. doi: 10.1002/jor.23794 29091320

[56] Hasegawa K Saga R Takahashi R Fukui R Chiba M Okumura K. 4-Methylumbelliferone Inhibits Clonogenic Potency by Suppressing High Molecular Weight-Hyaluronan in Fibrosarcoma Cells. Oncol Lett (2020) 19(4):2801–8. doi: 10.3892/ol.2020.11370 PMC7068617 32218833

[57] Saga R Matsuya Y Takahashi R Hasegawa K Date H Hosokawa Y. 4-Methylumbelliferone Administration Enhances Radiosensitivity of Human Fibrosarcoma by Intercellular Communication. Sci Rep (2021) 11(1):8258. doi: 10.1038/s41598-021-87850-3 33859324 PMC8050271

[58] Kudo D Kon A Yoshihara S Kakizaki I Sasaki M Endo M. Effect of a Hyaluronan Synthase Suppressor, 4-Methylumbelliferone, on B16F-10 Melanoma Cell Adhesion and Locomotion. Biochem Biophys Res Commun (2004) 321(4):783–7. doi: 10.1016/j.bbrc.2004.07.041 15358095

[59] Yoshihara S Kon A Kudo D Nakazawa H Kakizaki I Sasaki M. A Hyaluronan Synthase Suppressor, 4-Methylumbelliferone, Inhibits Liver Metastasis of Melanoma Cells. (2005) 579(12):2722–6. doi: 10.1016/j.febslet.2005.03.079 15862315

[60] Abildgaard C Rizza S Christiansen H Schmidt S Dahl C Abdul-Al A. Screening of Metabolic Modulators Identifies New Strategies to Target Metabolic Reprogramming in Melanoma. Sci Rep (2021) 11(1):4390. doi: 10.1038/s41598-021-83796-8 33623106 PMC7902673

[61] Ban H Uchakina O McKallip RJ. Hyaluronic Acid Inhibitor 4-Methylumbelliferone Activates the Intrinsic Apoptosis Pathway in K562 Chronic Myelogenous Leukemia Cells. Anticancer Res (2015) 35(10). 26408682

[62] Uchakina ON Ban H McKallip RJ. Targeting Hyaluronic Acid Production for the Treatment of Leukemia: Treatment With 4-Methylumbelliferone Leads to Induction of MAPK-Mediated Apoptosis in K562 Leukemia. Leuk Res (2013) 37(10):1294–301. doi: 10.1016/j.leukres.2013.07.009 23876826

[63] Uchakina ON Ban H Hostetler BJ McKallip RJ. Inhibition of Hyaluronic Acid Formation Sensitizes Chronic Myelogenous Leukemia to Treatment With Doxorubicin. Glycobiology (2016) 26(11):1171–9. doi: 10.1093/glycob/cww064 27261196

[64] Lompardía SL Papademetrio DL Mascaró M Del Carmen Álvarez EM Hajos SE. Human Leukemic Cell Lines Synthesize Hyaluronan to Avoid Senescence and Resist Chemotherapy. Glycobiology (2013) 23(12):1463–76. doi: 10.1093/glycob/cwt074 24013961

[65] Pibuel MA Poodts D Díaz M Hajos SE Lompardía SL. The Scrambled Story Between Hyaluronan and Glioblastoma. J Biol Chem (2021) 296:100549. doi: 10.1016/j.jbc.2021.100549. 33744285 PMC8050860

[66] Pibuel MA Díaz M Molinari Y Poodts D Silvestroff L Lompardía SL. 4-Methylumbelliferone as a Potent and Selective Antitumor Drug on a Glioblastoma Model. Glycobiology (2021) 31(1):29–43. doi: 10.1093/glycob/cwaa046 32472122

[67] Yan T Chen X Zhan H Yao P Wang N Yang H. Interfering With Hyaluronic Acid Metabolism Suppresses Glioma Cell Proliferation by Regulating Autophagy. Cell Death Dis (2021) 12(5):486. doi: 10.1038/s41419-021-03747-z 33986244 PMC8119697

[68] Winawer SJ Fletcher RH Miller L Godlee F Stolar MH Mulrow CD. Colorectal Cancer Screening: Clinical Guidelines and Rationale. Gastroenterology (1997) 112(2):594–642. doi: 10.1053/gast.1997.v112.agast970594 9024315

[69] Heffler M M. Golubovskaya V Conroy J Liu S Wang D G. Cance W. FAK and HAS Inhibition Synergistically Decrease Colon Cancer Cell Viability and Affect Expression of Critical Genes. Anticancer Agents Med Chem (2013) 13(4):584–94. doi: 10.2174/1871520611313040008 PMC3625516 22934709

[70] Wang TP Pan YR Fu CY Chang HY. Down-Regulation of UDP-Glucose Dehydrogenase Affects Glycosaminoglycans Synthesis and Motility in HCT-8 Colorectal Carcinoma Cells. Exp Cell Res (2010) 316(17):2893–902. doi: 10.1016/j.yexcr.2010.07.017 20691680

[71] Fries H Elsässer HP Mahlbacher V Kern HF Neumann K. Localisation of Hyaluronate (HA) in Primary Tumors and Nude Mouse Xenografts of Human Pancreatic Carcinomas Using a Biotinylated HA-Binding Protein. Virchows Arch (1994) 424(1):7–12. doi: 10.1007/BF00197386 7526947

[72] Suto A Kudo D Yoshida E Nagase H Suto S Mimura J. Increase of Tumor Infiltrating γδ T-Cells in Pancreatic Ductal Adenocarcinoma Through Remodeling of the Extracellular Matrix by a Hyaluronan Synthesis Suppressor, 4-Methylumbelliferone. Pancreas (2019) 48(2):292–8. doi: 10.1097/MPA.0000000000001211 30589828

[73] National Cancer Insitute. Cancer Stat Facts: Cancer of Any Site. National Cancer Institute. Available at: https://seer.cancer.gov/statfacts/html/all.html (Accesed May 10, 2021).

[74] Kato N Shibata K Uchigasaki S Kurose A. Relation Between Hyaluronan Synthesis and Cell Morphology in Ovarian Clear Cell Carcinomas. Pathol Int (2016) 66(4):218–23. doi: 10.1111/pin.12405 27017153

[75] Karalis TT Heldin P Vynios DH Neill T Buraschi S Iozzo RV. Tumor-Suppressive Functions of 4-MU on Breast Cancer Cells of Different ER Status: Regulation of Hyaluronan/HAS2/CD44 and Specific Matrix Effectors. Matrix Biol (2019) 78–79(2017):118–38. doi: 10.1016/j.matbio.2018.04.007 29673760

[76] Sevic I Spinelli FM Cantero MJ Reszegi A Kovalszky I García MG. The Role of the Tumor Microenvironment in the Development and Progression of Hepatocellular Carcinoma. In: Hepatocell Carcinoma Codon Publications (2019) p:29–45. doi: 10.15586/hepatocellularcarcinoma.2019.ch2 31664802

[77] Loomba R Adams LA. Advances in non-Invasive Assessment of Hepatic Fibrosis. Gut (2020) 69(7):1343–52. doi: 10.1136/gutjnl-2018-317593 PMC7945956 32066623

[78] Piccioni F Malvicini M Garcia MG Rodriguez A Atorrasagasti C Kippes N. Antitumor Effects of Hyaluronic Acid Inhibitor 4-Methylumbelliferone in an Orthotopic Hepatocellular Carcinoma Model in Mice. Glycobiology (2012) 22(3):400–10. doi: 10.1093/glycob/cwr158 22038477

[79] Mikami K Endo T Sawada N Igarashi G Kimura M Sakuraba H. Inhibition of Systemic Hyaluronan Synthesis Exacerbates Murine Hepatic Carcinogenesis. In Vivo (Brooklyn) (2018) 32(2):273–8. doi: 10.21873/invivo.11234 PMC5905194 29475909

[80] Takasugi M Firsanov D Tombline G Ning H Ablaeva J Seluanov A. Naked Mole-Rat Very-High-Molecular-Mass Hyaluronan Exhibits Superior Cytoprotective Properties. Nat Commun (2020) 11(1):2376. doi: 10.1038/s41467-020-16050-w 32398747 PMC7217962

[81] Nishida Y Knudson W Knudson CB Ishiguro N. Antisense Inhibition of Hyaluronan Synthase-2 in Human Osteosarcoma Cells Inhibits Hyaluronan Retention and Tumorigenicity. Exp Cell Res (2005) 307(1):194–203. doi: 10.1016/j.yexcr.2005.03.026 15922739 PMC3182490

[82] Suzuki Y Nishida Y Naruse T Gemba T Ishiguro N. Pericellular Matrix Formation Alters the Efficiency of Intracellular Uptake of Oligonucleotides in Osteosarcoma Cells. J Surg Res (2009) 152(1):148–56. doi: 10.1016/j.jss.2008.02.037 18533189

[83] Hosono K Nishida Y Knudson W Knudson CB Naruse T Suzuki Y. Hyaluronan Oligosaccharides Inhibit Tumorigenicity of Osteosarcoma Cell Lines MG-63 and LM-8 *In Vitro* and *In Vivo via* Perturbation of Hyaluronan-Rich Pericellular Matrix of the Cells. Am J Pathol (2007) 171(1):274–86. doi: 10.2353/ajpath.2007.060828 PMC1941604 17591972

[84] Gelderblom H Hogendoorn PCW Dijkstra SD van Rijswijk CS Krol AD Taminiau AHM. The Clinical Approach Towards Chondrosarcoma. Oncologist (2008) 13(3):320–9. doi: 10.1634/theoncologist.2007-0237 18378543

[85] Van oosterwijk JG Herpers B Meijer D Briaire-de bruijn IH Cleton-jansen AM Gelderblom H. Restoration of Chemosensitivity for Doxorubicin and Cisplatin in Chondrosarcoma *In Vitro*: BCL-2 Family Members Cause Chemoresistance. Ann Oncol (2012) 23(6):1617–26. doi: 10.1093/annonc/mdr512 22112972

[86] Saga R Monzen S Chiba M Yoshino H Nakamura T Hosokawa Y. Anti-Tumor and Anti-Invasion Effects of a Combination of 4-Methylumbelliferone and Ionizing Radiation in Human Fibrosarcoma Cells. Oncol Lett (2017) 13(1):410–6. doi: 10.3892/ol.2016.5385 PMC5245070 28123575

[87] Saga R Hasegawa K Murata K Chiba M Nakamura T Okumura K. Regulation of Radiosensitivity by 4-Methylumbelliferone *via* the Suppression of Interleukin-1 in Fibrosarcoma Cells. Oncol Lett (2019) 17(3):3555–61. doi: 10.3892/ol.2019.9990 PMC6396223 30867797

[88] Zagars GK Ballo MT Pisters PWT Pollock RE Patel SR Benjamin RS. Prognostic Factors for Patients With Localized Soft-Tissue Sarcoma Treated With Conservation Surgery and Radiation Therapy: An Analysis of 1225 Patients. Cancer (2003) 97(10):2530–43. doi: 10.1002/cncr.11365 12733153

[89] Eggermont AMM Spatz A Robert C. Seminar Cutaneous Melanoma. Lancet (2014) 383(9919):816–27. doi: 10.1016/S0140-6736(13)60802-8 24054424

[90] Bai X Flaherty KT. Targeted and Immunotherapies in BRAF Mutant Melanoma: Where We Stand and What to Expect. Br J Dermatol (2020)185(2):253–62. doi: 10.1111/bjd.19394 32652567

[91] Edward M Quinn JA Pasonen-Seppänen SM McCann BA Tammi RH. 4-Methylumbelliferone Inhibits Tumour Cell Growth and the Activation of Stromal Hyaluronan Synthesis by Melanoma Cell-Derived Factors. Br J Dermatol (2010) 162(6):1224–32. doi: 10.1111/j.1365-2133.2010.09699.x 20163414

[92] André-Grégoire G Gavard J. Spitting Out the Demons: Extracellular Vesicles in Glioblastoma. Cell Adhesion Migration (2017) 11(2):164–72. doi: 10.1080/19336918.2016.1247145. PMC5351723 27736300

[93] Wiranowska M Ladd S Moscinski LC Hill B Haller E Mikecz K. Modulation of Hyaluronan Production by CD44 Positive Glioma Cells. Int J Cancer (2010) 127(3):532–42. doi: 10.1002/ijc.25085 PMC3962665 19957333

[94] Kim Y Kumar S. CD44-Mediated Adhesion to Hyaluronic Acid Contributes to Mechanosensing and Invasive Motility. Mol Cancer Res (2014) 12(10):1416–29. doi: 10.1158/1541-7786.MCR-13-0629 PMC4201971 24962319

[95] Enegd B King JAJ Stylli S Paradiso L Kaye AH Novak U. Overexpression of Hyaluronan Synthase-2 Reduces the Tumorigenic Potential of Glioma Cells Lacking Hyaluronidase Activity. Neurosurgery (2002) 50(6):1311–8. doi: 10.1227/00006123-200206000-00023 12015850

[96] Mueller AM Yoon BH Sadiq SA. Inhibition of Hyaluronan Synthesis Protects Against Central Nervous System (CNS) Autoimmunity and Increases CXCL12 Expression in the Inflamed CNS. J Biol Chem (2014) 289(33):22888–99. doi: 10.1074/jbc.M114.559583 PMC4132791 24973214

[97] Institute NNC. Cancer Facts & Figures 2020. CA Cancer J Clin (2020). Available at: https://www.cancer.org/research/cancer-facts-statistics/all-cancer-facts-figures/cancer-facts-figures-2020.html (Accesed May 10, 2021).

[98] Jabbour E Kantarjian H. Chronic Myeloid Leukemia: 2018 Update on Diagnosis, Therapy and Monitoring. Am J Hematol (2018) 93(3):442–59. doi: 10.1002/ajh.25011 29411417

[99] Jain RK. Normalizing Tumor Microenvironment to Treat Cancer: Bench to Bedside to Biomarkers. In: Journal of Clinical Oncology. Am Soc Clin Oncol (2013) 31(17):2205–18. doi: 10.1200/JCO.2012.46.3653 PMC3731977 23669226

[100] Casey T Bond J Tighe S Hunter T Lintault L Patel O. Molecular Signatures Suggest a Major Role for Stromal Cells in Development of Invasive Breast Cancer. Breast Cancer Res Treat (2009) 114(1):47–62. doi: 10.1007/s10549-008-9982-8 18373191

[101] Naba A Clauser KR Hoersch S Liu H Carr SA Hynes RO. The Matrisome: *In Silico* Definition and *In Vivo* Characterization by Proteomics of Normal and Tumor Extracellular Matrices. Mol Cell Proteomics (2012) 11(4):M111.014647. doi: 10.1074/mcp.M111.014647 PMC3322572 22159717

[102] Knudson W Biswas C Toole BP. Interactions Between Human Tumor Cells and Fibroblasts Stimulate Hyaluronate Synthesis. Proc Natl Acad Sci USA (1984) 81(21):6767–71. doi: 10.1073/pnas.81.21.6767 PMC392012 6593727

[103] Asplund T Versnel MA Laurent TC Heldin P. Human Mesothelioma Cells Produce Factors That Stimulate the Production of Hyaluronan by Mesothelial Cells and Fibroblasts. Cancer Res (1993) 53(2). 8417831

[104] Pirinen R Tammi R Tammi M Hirvikoski P Parkkinen JJ Johansson R. Prognostic Value of Hyaluronan Expression in Non-Small-Cell Lung Cancer: Increased Stromal Expression Indicates Unfavorable Outcome in Patients With Adenocarcinoma. Int J Cancer (2001) 95(1):12–7. doi: 10.1002/1097-0215(20010120)95:1<12::AID-IJC1002>3.0.CO;2-E 11241304

[105] Liu J Geng X Hou J Wu G. New Insights Into M1/M2 Macrophages: Key Modulators in Cancer Progression. Cancer Cell Int (2021) 21(1):389. doi: 10.1186/s12935-021-02089-2. 34289846 PMC8296555

[106] Bingle L Brown NJ Lewis CE. The Role of Tumour-Associated Macrophages in Tumour Progression: Implications for New Anticancer Therapies. J Pathol (2002) 196(3):254–65. doi: 10.1002/path.1027 11857487

[107] McKee CM Penno MB Cowman M Burdick MD Strieter RM Bao C. Hyaluronan (HA) Fragments Induce Chemokine Gene Expression in Alveolar Macrophages: The Role of HA Size and CD44. J Clin Invest (1996) 98(10):2403–13. doi: 10.1172/JCI119054 PMC507693 8941660

[108] Noble PW Lake FR Henson PM Riches DWH. Hyaluronate Activation of CD44 Induces Insulin-Like Growth Factor-1 Expression by a Tumor Necrosis Factor-α-Dependent Mechanism in Murine Macrophages. J Clin Invest (1993) 91(6):2368–77. doi: 10.1172/JCI116469 PMC443294 8514850

[109] Yamawaki H Hirohata S Miyoshi T Takahashi K Ogawa H Shinohata R. Hyaluronan Receptors Involved in Cytokine Induction in Monocytes. Glycobiology (2009) 19(1):83–92. doi: 10.1093/glycob/cwn109 18854367

[110] Scheibner KA Lutz MA Boodoo S Fenton MJ Powell JD Horton MR. Hyaluronan Fragments Act as an Endogenous Danger Signal by Engaging Tlr2. J Immunol (2006) 177(2):1272–81. doi: 10.4049/jimmunol.177.2.1272 16818787

[111] Jiang D Liang J Noble PW. Hyaluronan as an Immune Regulator in Human Diseases. Physiol Rev (2011) 91(1):221–64. doi: 10.1152/physrev.00052.2009 PMC3051404 21248167

[112] Bollyky PL Lord JD Masewicz SA Evanko SP Buckner JH Wight TN. Cutting Edge: High Molecular Weight Hyaluronan Promotes the Suppressive Effects of CD4 + CD25 + Regulatory T Cells. J Immunol (2007) 179(2):744–7. doi: 10.4049/jimmunol.179.2.744 17617562

[113] Spinelli FM Vitale DL Icardi A Caon I Brandone A Giannoni P. Hyaluronan Preconditioning of Monocytes/Macrophages Affects Their Angiogenic Behavior and Regulation of TSG-6 Expression in a Tumor Type-Specific Manner. FEBS J (2019) 286(17):3433–49. doi: 10.1111/febs.14871 31044513

[114] Itatani Y Kawada K Yamamoto T Sakai Y. Resistance to Anti-Angiogenic Therapy in Cancer-Alterations to Anti-VEGF Pathway. Int J Mol Sci (2018) 19(4):1232. doi: 10.3390/ijms19041232 PMC5979390 29670046

[115] Karamanos NK Theocharis AD Piperigkou Z Manou D Passi A Skandalis SS. A Guide to the Composition and Functions of the Extracellular Matrix. FEBS J (2021). doi: 10.1111/febs.15776 33605520

[116] Provenzano PP Hingorani SR. Hyaluronan, Fluid Pressure, and Stromal Resistance in Pancreas Cancer. Br J Cancer Br J Cancer (2013) 108:1–8. doi: 10.1038/bjc.2012.569 23299539 PMC3553539

[117] Dufort CC DelGiorno KE Carlson MA Osgood RJ Zhao C Huang Z. Interstitial Pressure in Pancreatic Ductal Adenocarcinoma Is Dominated by a Gel-Fluid Phase. Biophys J (2016) 110(9):2106–19. doi: 10.1016/j.bpj.2016.03.040 PMC4939548 27166818

[118] Jacobetz MA Chan DS Neesse A Bapiro TE Cook N Frese KK. Hyaluronan Impairs Vascular Funct0ion and Drug Delivery in a Mouse Model of Pancreatic Cancer. Gut (2013) 62(1):112–20. doi: 10.1136/gutjnl-2012-302529 PMC3551211 22466618

[119] Provenzano PP Cuevas C Chang AE Goel VK Von Hoff DD Hingorani SR. Enzymatic Targeting of the Stroma Ablates Physical Barriers to Treatment of Pancreatic Ductal Adenocarcinoma. Cancer Cell (2012) 21(3):418–29. doi: 10.1016/j.ccr.2012.01.007 PMC3371414 22439937

[120] Ariffin AB Forde PF Jahangeer S Soden DM Hinchion J. Releasing Pressure in Tumors: What do We Know So Far and Where Do We Go From Here a Review. Cancer Res Am Assoc Cancer Res Inc (2014) 74:2655–62. doi: 10.1158/0008-5472.CAN-13-3696 24778418

[121] Infante JR Korn RL Rosen LS Lorusso P Dychter SS Zhu J. Phase 1 Trials of PEGylated Recombinant Human Hyaluronidase PH20 in Patients With Advanced Solid Tumours. Br J Cancer (2018) 118(2):153–61. doi: 10.1038/bjc.2017.327 PMC5785735 28949957

[122] Hakim N Patel R Devoe C Saif MW. Why HALO 301 Failed and Implications for Treatment of Pancreatic Cancer. Pancreas – Open J (2019) 3(1):e1–4. doi: 10.17140/POJ-3-e010 PMC7003617 32030361

[123] Keller KE Sun YY Vranka JA Hayashi L Acott TS. Inhibition of Hyaluronan Synthesis Reduces Versican and Fibronectin Levels in Trabecular Meshwork Cells. PloS One (2012) 7(11):48523. doi: 10.1371/journal.pone.0048523 PMC3489675 23139787

[124] Andreichenko IN Tsitrina AA Fokin AV Gabdulkhakova AI Maltsev DI Perelman GS. 4-Methylumbelliferone Prevents Liver Fibrosis by Affecting Hyaluronan Deposition, FSTL1 Expression and Cell Localization. Int J Mol Sci (2019) 20(24):6301. doi: 10.3390/ijms20246301 PMC6941058 31847129

[125] Rilla K Pasonen-Seppänen S Rieppo J Tammi M Tammi R. The Hyaluronan Synthesis Inhibitor 4-Methylumbelliferone Prevents Keratinocyte Activation and Epidermal Hyperproliferation Induced by Epidermal Growth. J Invest Dermatol (2004) 123(4):708–14. doi: 10.1111/j.0022-202X.2004.23409.x 15373776

[126] Nakamura T Ishikawa T Nanashima N Miura T Nozaka H Nakaoka R. 4-Methylumbelliferone Induces the Expression of Membrane Type 1-Matrix Metalloproteinase in Cultured Human Skin Fibroblasts. Biochem Biophys Res Commun (2002) 298(5):646–50. doi: 10.1016/S0006-291X(02)02516-0 12419303

[127] Nakamura R Kuwabara H Yoneda M Yoshihara S Ishikawa T Miura T. Suppression of Matrix Metalloproteinase-9 by 4-Methylumbelliferone. Cell Biol Int (2007) 31(9):1022–6. doi: 10.1016/j.cellbi.2007.03.016 17470403

[128] Hobarth K Maier U Marberger M. Topical Chemoprophylaxis of Superficial Bladder Cancer With Mitomycin C and Adjuvant Hyaluronidase. Eur Urol (1992) 21(3):206–10. doi: 10.1159/000474839 1499626

[129] Ruponen M Honkakoski P Tammi M Urtti A. Cell-Surface Glycosaminoglycans Inhibit Cation-Mediated Gene Transfer. J Gene Med (2004) 6(4):405–14. doi: 10.1002/jgm.522 15079815

[130] Vitale DL Caon I Parnigoni A Sevic I Spinelli FM Icardi A. Initial Identification of UDP-Glucose Dehydrogenase as a Prognostic Marker in Breast Cancer Patients, Which Facilitates Epirubicin Resistance and Regulates Hyaluronan Synthesis in MDA-MB-231 Cells. Biomolecules (2021) 11(2):1–31. doi: 10.3390/biom11020246 PMC7914570 33572239

[131] Miyamoto H Murakami T Tsuchida K Sugino H Miyake H Tashiro S. Tumor-Stroma Interaction of Human Pancreatic Cancer: Acquired Resistance to Anticancer Drugs and Proliferation Regulation Is Dependent on Extracellular Matrix Proteins. Pancreas (2004) 28(1):38–44. doi: 10.1097/00006676-200401000-00006 14707728

[132] Lokman NA Price ZK Hawkins EK Macpherson AM Oehler MK Ricciardelli C. 4-Methylumbelliferone Inhibits Cancer Stem Cell Activation and Overcomes Chemoresistance in Ovarian Cancer. Cancers (Basel) (2019) 11(8):1187. doi: 10.3390/cancers11081187 PMC6721459 31443261

[133] Wirsching H-G Galanis E. Chapter 23 – Glioblastoma. Handbook of Clinical Neurology (2016) 134:381–97. doi: 10.1016/B978-0-12-802997-8.00023-2. 26948367

[134] Hall A Meyle KD Lange MK Klima M Sanderhoff M Dahl C. Dysfunctional Oxidative Phosphorylation Makes Malignant Melanoma Cells Addicted to Glycolysis Driven by the V600EBRAF Oncogene. Oncotarget (2013) 4(4):584–99. doi: 10.18632/oncotarget.965 PMC3720606 23603840

[135] Haq R Fisher DE Widlund HR. Molecular Pathways: BRAF Induces Bioenergetic Adaptation by Attenuating Oxidative Phosphorylation. Clin Cancer Res (2014) 20(9):2257–63. doi: 10.1158/1078-0432.CCR-13-0898 PMC4008642 24610826

[136] Haq R Shoag J Andreu-Perez P Yokoyama S Edelman H Rowe GC. Oncogenic BRAF Regulates Oxidative Metabolism *via* PGC1α and MITF. Cancer Cell (2013) 23(3):302–15. doi: 10.1016/j.ccr.2013.02.003 PMC3635826 23477830

[137] Vazquez F Lim JH Chim H Bhalla K Girnun G Pierce K. Pgc1α Expression Defines a Subset of Human Melanoma Tumors With Increased Mitochondrial Capacity and Resistance to Oxidative Stress. Cancer Cell (2013) 23(3):287–301. doi: 10.1016/j.ccr.2012.11.020 23416000 PMC3708305

[138] Sleire L Førde-Tislevoll HE Netland IA Leiss L Skeie BS Enger PØ. Drug Repurposing in Cancer. Pharmacol Res (2017) 124:74–91. doi: 10.1016/j.phrs.2017.07.013 28712971

[139] Singh TU Parida S Lingaraju MC Kesavan M Kumar D Singh RK. Drug Repurposing Approach to Fight COVID-19. Pharmacol Rep (2020) 72(6):1479–508. doi: 10.1007/s43440-020-00155-6 PMC7474498 32889701

[140] Kobayashi N Miyoshi S Mikami T Koyama H Kitazawa M Takeoka M. Hyaluronan Deficiency in Tumor Stroma Impairs Macrophage Trafficking and Tumor Neovascularization. Cancer Res (2010) 70(18):7073–83. doi: 10.1158/0008-5472.CAN-09-4687 20823158

[141] Kuipers HF Nagy N Ruppert SM Sunkari VG Marshall PL Gebe JA. The Pharmacokinetics and Dosing of Oral 4-Methylumbelliferone for Inhibition of Hyaluronan Synthesis in Mice. Clin Exp Immunol (2016) 185(3):372–81. doi: 10.1111/cei.12815 PMC4991518 27218304

[142] Nagy N Gurevich I Kuipers HF Ruppert SM Marshall PL Xie BJ. 4-Methylumbelliferyl Glucuronide Contributes to Hyaluronan Synthesis Inhibition. J Biol Chem (2019) 294(19):7864–77. doi: 10.1074/jbc.RA118.006166 PMC6514619 30914479

